# Genome-wide DNA methylation patterns for indicators of liver steatosis: a longitudinal multiomic study

**DOI:** 10.1186/s13148-025-02037-1

**Published:** 2026-01-03

**Authors:** Jo Ciantar, Sonja Rajić, Daria Kostiniuk, Ella Raulamo, Noora Kartiosuo, Liye Lai, Pashupati P. Mishra, Leo-Pekka Lyytikäinen, Marcus E. Kleber, Suvi Rovio, Juha Mykkänen, Katja Pahkala, Annette Peters, Juliane Winkelmann, Winfried März, Mika Kähönen, Olli Raitakari, Terho Lehtimäki, Melanie Waldenberger, Saara Marttila, Emma Raitoharju

**Affiliations:** 1 Molecular Epidemiology, Faculty of Medicine and Health Technology, Tampere University, Tampere, Finland; 2 Department of Mathematics and Statistics, University of Turku, Turku, Finland; 3 Centre for Population Health Research, University of Turku and Turku University Hospital, Turku, Finland; 4 Research Centre of Applied and Preventive Cardiovascular Medicine, University of Turku, Turku, Finland; 5 Research Unit Molecular Epidemiology, Helmholtz Zentrum München, German Research Center for Environmental Health, Neuherberg, Germany; 6 Faculty of MedicinePettenkofer School of Public HealthInstitute for Medical Information Processing, Biometry, and Epidemiology (IBE), Ludwig Maximilians University, Munich, Germany; 7 Department of Clinical Chemistry Faculty of Medicine and Health Technology, Tampere University, Tampere, Finland; 8 Medical Faculty Mannheim, Department of Cardiology, Angiology, Hemostaseology and Medical Intensive Care, University Medical Center Mannheim, Heidelberg University, Mannheim, Germany; 9 SYNLAB MVZ Humangenetik Mannheim, Mannheim, Germany; 10 Department of Public Health, University of Turku and Turku University Hospital, Turku, Finland; 11 Unit for Health and Physical Activity, Paavo Nurmi Centre, University of Turku, Turku, Finland; 12 Epidemiology, Institute for Medical Information Processing, Biometry, and Epidemiology–IBE, Ludwig-Maximilians University of Munich, Munich, Germany; 13 German Center for Diabetes Research (DZD) Munich, Neuherberg, Germany; 14 Computational Health Center, Helmholtz Munich, Institute of Neurogenomics, Neuherberg, Germany; 15 Synlab Academy, SYNLAB Holding Deutschland GmbH, Augsburg , Mannheim, Germany; 16 Department of Clinical Physiology, Tampere University Hospital and Faculty of Medicine and Health Technology, Tampere University, Tampere, Finland; 17 Department of Clinical Physiology and Nuclear Medicine, Turku University Hospital, Turku, Finland; 18 Fimlab Laboratories, Tampere, Finland; 19 Faculty of Medicine and Health Technology, Finnish Cardiovascular Research Center Tampere, Tampere University, Tampere, Finland; 20 Helmholtz Zentrum München, German Research Center for Environmental Health, Institute of Epidemiology, Neuherberg, Germany; 21 German Research Center for Cardiovascular Disease (DZHK), Partner Site Munich Heart Alliance, Munich, Germany; 22 Gerontology Research Center, Tampere University, Tampere, Finland; 23 Tays Research Services,, Wellbeing Services County of Pirkanmaa, Tampere University Hospital, Tampere, Finland

**Keywords:** Metabolic dysfunction associated steatotic liver disease, DNA methylation, Longitudinal, Population cohort, Non-alcoholic fatty liver disease, GGT, Fatty liver index

## Abstract

**Background:**

To identify blood DNA methylation profiles related to liver steatosis, we performed an EWAS on the presence of ultrasonically-identified liver steatosis in the Young Finns Study (YFS) participants (n = 1529, 33–50y.), and on liver enzyme levels and fatty liver index (FLI) across three discovery cohorts: YFS, LURIC (n = 2371, 17–92y.) and KORA FF4 (n = 1872, 39–88y.). We further investigated the discovered associations across the longitudinal subset of YFS (n = 255), encompassing three follow-ups over 32 years, and the three-generational YFS-3G follow-up in 2018–2020. Finally, we examined the associations of the discovered CpGs with nearby genetic variation and whole blood expression of nearby genes.

**Results:**

In YFS, the methylation levels of cg06690548 (*SLC7A11*) were lower in individuals with liver steatosis (Δbeta = − 0.011, FDR = 0.004). Methylation of 9 CpGs associated with GGT and 23 CpGs with FLI in at least two of the discovery cohorts. Methylation at cg06690548 (*SLC7A11*) and the majority of the CpGs associating with GGT or FLI had the strongest association in the two oldest generations of YFS-3G follow-up (ages 43–59y. and 59–93y.), with minor or non-significant association in the youngest generation (ages 6–36y.). Discovered meQTLs for the CpGs did not modulate the association between the methylation levels and GGT or FLI. The expression of the nearby genes mediated only the association between cg06500161 (*ABCG1*) and cg20544516 (*SREBF1*) and FLI.

**Conclusions:**

Our findings highlight the association between the methylation levels of cg06690548 (*SLC7A11)* and liver steatosis, describe the dynamic relationship between whole blood DNA methylation and MASLD, and contribute to a deeper understanding of the pathophysiology of liver diseases.

**Supplementary Information:**

The online version contains supplementary material available at 10.1186/s13148-025-02037-1.

## Background

Steatotic liver disease is a spectrum of liver conditions with various aetiologies, ranging from mild fat infiltration to steatohepatitis, fibrosis and cirrhosis [1]. The most common subtype, known as non-alcoholic fatty liver disease (NAFLD), has recently been revised as metabolic dysfunction-associated steatotic liver disease (MASLD) to emphasise the central role of metabolic dysfunction in the pathogenesis of the disease [1]. MASLD is defined as over 5% liver fat content accompanied by at least one cardiovascular risk factor [1], and about 99% of patients diagnosed with NAFLD meet this criterion [2]. Affecting 25–40% of the global population, MASLD’s alarmingly increasing prevalence is contributing to the growing liver transplantation burden [3–5]. MASLD is the leading cause of chronic liver diseases and is a risk factor for cardiovascular diseases, overall mortality, and decreased quality of life [6, 7]. It is a reversible condition, making early identification and lifestyle interventions crucial to prevent its progression to chronic and severe liver diseases, such as steatohepatitis, cirrhosis, and hepatocellular carcinoma [3–5].

The increase in the prevalence of MASLD parallels with the rise in sedentary behaviour, obesity, insulin resistance, and metabolic syndrome, but the pathophysiology and contributing factors are not fully understood [8, 9]. Insulin resistance is a key component in MASLD development that increases de novo lipogenesis and enhances fatty acid transport from adipose tissue to the liver [10]. Similarly, obesity increases circulating free fatty acid levels contributing to MASLD development and progression [8]. While alcohol-related liver disease (ALD) and NAFLD have been considered distinct conditions since 1980, both share similar pathophysiological mechanisms [11–13]. To acknowledge this, the revised terminology also introduced “metabolic dysfunction and alcohol-associated liver disease” (metALD), capturing the coexistence of two distinct aetiologies without excluding their shared pathophysiology [1].

DNA methylation is a reversible epigenetic mechanism that regulates gene expression and can have phenotypic effects. It has been shown to mediate the adaptation to many environmental exposures, such as changes in nutritional status and metabolic diseases [14]. Liver biopsies of NAFLD patients exhibit significantly lower global DNA methylation levels in comparison to biopsies from obese controls, with methylation levels correlating with severity of NAFLD [15]. Furthermore, Ahrens et al. have reported changes in liver DNA methylation patterns in genes related to intermediary metabolism and insulin signalling, which also correlated with NAFLD severity [16].

Peripheral blood DNA methylation has been suggested to be a potential biomarker for liver steatosis [17]. Previous EWASes have shown that methylation levels in CpGs of several solute carriers (SLCs) and phosphoglycerate dehydrogenase (*PHGDH*) are robustly associated with gamma-glutamyltransferase (GGT) levels [18] and identified 22 CpGs, including several CpGs in SLCs and *PHGDH*, associated with NAFLD [19]. These studies have relied on a single discovery cohort (n < 1500), with the significant findings subsequently replicated in several smaller cohorts. Several studies have explored whether changes in DNA methylation profiles associated with liver steatosis are causal or precede the onset of metabolic diseases. Nano et al. linked solute carrier family 7 member 11 (*SLC7A11*) to the expression of lipid-associated genes and showed the causality of CpGs associated with GGT using Mendelian randomisation [18]. Ma et al. showed that some CpGs associated with NAFLD were predictive of the onset of type 2 diabetes (T2D) [19]. However, both studies measured DNA methylation at a single timepoint, making it impossible to investigate the dynamic changes in methylation profiles over time. To address this gap, Zeng et al. conducted a longitudinal analysis of peripheral blood DNA methylation and liver fat content in 96 individuals with approximately five-year follow-up. Their results indicated that DNA methylation in *SREBF1, ADBCG1, DHCR24, CTPT1A* and *LINC00649* may predict the change in liver fat content, but the associations between *SREBF1* and *ADBCG1* were largely influenced by body weight [20].

Here, we set to investigate the association between whole blood DNA methylation, measured with the Illumina EPIC array, and ultrasonically identified liver steatosis (MASLD and metALD) using the 2011 follow-up of the Young Finns Study (YFS, n = 1529). These findings were replicated across three generations in the 2018 YFS-3G follow-up, which included the original YFS cohort (G1, n = 1273), their parents (G0, n = 1342), and their offspring (G2, n = 1309). We also investigated whether the methylation levels of CpGs that were associated with liver steatosis predicted its presence in 2018 prospectively in 2011 and 1986 in a subset of the YFS participants with longitudinal DNA methylation data (n = 255). In addition, we aimed to discover CpGs associated with three separate liver enzymes and fatty liver index (FLI) in at least two of the discovery cohorts–YFS 2011 follow-up (n = 1529), German cohorts Cooperative Health Research in the Region of Augsburg (KORA, n = 1872), and the LUdwigshafen RIsk and Cardiovascular Health study (LURIC, n = 2371)—and validate these associations in the three-generational YFS-3G 2018 follow-up. Finally, we investigated whether methylation quantitative trait loci (meQTLs) influenced the methylation levels of the discovered CpGs and whether the methylation levels were associated with the expression of nearby genes in whole blood.

## Materials and methods

**Fig. 1:**
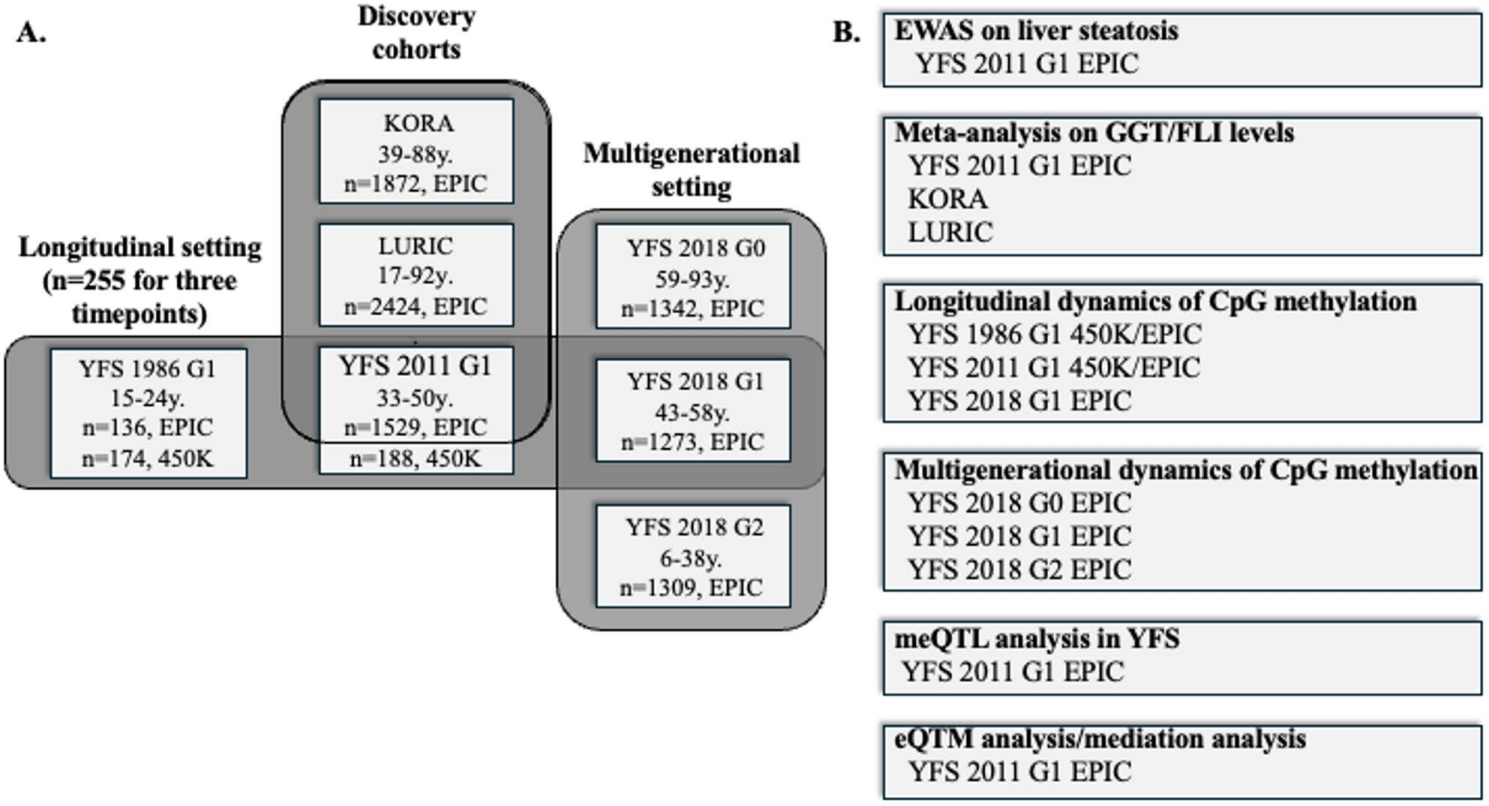
**A** Cohorts and **B** study settings utilised in the study. Out of the discovery cohorts, the information on the presence of steatotic liver was available only from the YFS 2011 and 2018 follow-ups, while liver enzyme levels and FLI were available also from KORA and LURIC. The longitudinal study setting is comprised of 255 individuals from whom we have DNA methylation data available from the three follow-ups spanning over 30 years, while in the multigenerational setting we utilise DNA methylation data available from the follow-up cohort (G1) and their parents (G0) and children (G2), all of whom participated in the 2018 follow-up of YFS. Abbreviations: G = generation, EWAS = Epigenome wide association study, GGT = gamma glutamyl transferase, FLI = fatty liver index, meQTL = methylation quantitative trait loci, eQTM = expression quantitative trait methylation

Cohorts and different analysis settings utilised are described in Fig. 1. Throughout the text and tables hg19/hg37 genetic coordinates are utilized.

### YFS

The YFS is a multicentre follow-up study on cardiovascular risk from childhood to adulthood in Finland. The study was launched in 1980, with 3596 children and adolescents (aged 3–18 years) participating in the baseline study [21]. Participants have been followed through several examinations, including comprehensive risk factor assessments with major follow-ups in 1986, 2001, 2007, 2011 and 2018–2020. The 40-year follow-up was conducted in 2018 and expanded to include parents (G0) and children (G2) of the original YFS participants (G1) [22].

DNA methylation was successfully profiled for 311 study participants in the 1986 follow-up, 1714 in the 2011 follow-up, and 1311 in the 2018 follow-up, with 255 original study participants having DNA methylation data available for all three time points. In addition, DNA methylation data was available from the 2018 follow-up for 1343 parents (G0) and 1309 children (G2). See Supplementary Table 1 for the demographics of the utilised populations.

### KORA

The KORA FF4 study is the second follow-up of the KORA S4 study, conducted in 2013–2014. Of the 4261 participants of the S4 baseline study, 2279 also participated in the FF4 14-year follow-up study. In both follow-up surveys, participants completed a lifestyle questionnaire and underwent standardised examinations including blood samples collection, as described previously [23, 24]. DNA methylation profiling was successful for 1872 participants of the KORA FF4 study (39–88y.).

### LURIC

LURIC is a patient cohort of 3316 German individuals aged 17–92 years, who underwent coronary angiography between 1997 and 2000 and have since been followed up on non-fatal events after five years, and on all-cause and cause-specific mortality after 10 years [25]. DNA methylation profiling was successful for 2371 participants of the LURIC study.

### DNA methylation

For the DNA methylation analysis of the YFS cohort, leukocyte DNA was obtained from EDTA blood samples collected during the 1986 and 2011 follow-ups using a Wizard® Genomic DNA Purification Kit (Promega Corporation, Madison, WI, USA). Samples in the 2018 follow-up were processed using the Perkin Elmer Chemagic (CMG-1074) according to the manufacturer’s instructions, except for utilizing modified binding buffer 2 with a subset of the samples.

In YFS, 181 of the 1986 samples and 188 of the 2011 samples were analyzed with Illumina Infinium HumanMethylation450 BeadChips. The 1986 samples were processed at the Helmholtz Zentrum in Munich, Germany, and the 2011 samples in the Core Facility at the Institute of Molecular Medicine Finland (FIMM), University of Helsinki, according to the protocol by Illumina, as described earlier [26]. 130 of the YFS 1986 samples and 1526 of the YFS 2011 samples were analyzed with the Illumina Infinium MethylationEPIC v1 BeadChip at Helmholtz Zentrum Munich, Germany as described earlier [27]. Samples were applied to the arrays in a randomised order. Aliquots of 1 μg of DNA were subjected to bisulphite conversion, and a 4 μl aliquot of bisulphite-converted DNA was subjected to whole-genome amplification, followed by enzymatic fragmentation and hybridization onto an Illumina Infinium MethylationEPIC BeadChip. The arrays were scanned with the iScan reader (Illumina). All pre-processing steps were performed using functions implemented in the *minfi* R/Bioconductor package [28]. Cross-reactive probes [29, 30] and probes with SNPs given by minfi were removed. For discovery analysis 769,683 probes from 1526 samples with profiling done with EPIC v1 were utilized.

For the YFS 2018–2020 follow-up, blood DNA methylation profiling was performed for 3963 participants, spanning all three generations (G0 n = 1343, G1 = 1311, and G2 n = 1309). In short, methylation profiling was performed with the Illumina Infinium MethylationEPIC BeadChip v1 and v2 at Helmholtz Zentrum, Munich, Germany. Samples were applied to the arrays in a randomised order. Aliquots of 1 μg of DNA were subjected to bisulphite conversion, and a 4 μl aliquot of bisulphite-converted DNA was subjected to whole-genome amplification, followed by enzymatic fragmentation and hybridization onto an Illumina Infinium MethylationEPIC BeadChip (version 1 and 2). The arrays were scanned with the iScan reader (Illumina). Background subtraction was performed on all probe values using the bgcorrect.illumina function in the *minfi* package. Probes with a detection *p*-value > 10e–16 and samples with a sample call rate of more than 95% were filtered out. Autosomal probes were separated into six groups by probe-type and quantile normalization was performed separately for each group. Samples for which the actual sex did not match the predicted sex were excluded and quality control was performed on the data. All pre-processing steps were performed using functions implemented in the *minfi*, *sesame* or *limma* R/Bioconductor package. For 255 individuals with available methylation data from all three YFS follow-ups, data from 1986 and 2011 was renormalized with *sesame* to enable longitudinal analysis.

For **KORA** genome-wide DNA methylation was performed using the Illumina Infinium MethylationEPIC v1 BeadChip® for 1,928 KORA FF4 samples. Genomic DNA (750 ng) was bisulphite-converted using the EZ-96 DNA Methylation Kit in two separate batches (n = 488, n = 1440). A subsequent methylation analysis was performed on an Illumina iScan platform according to standard protocols provided by Illumina. GenomeStudio software (version 2011.1) with Methylation Module v1.9.0 was used for an initial quality control of assay performance and for the generation of methylation data export files. Further quality control and preprocessing of the data were performed in R v3.5.1 with the package *minfi* v1.28.3, and following primarily the CPACOR pipeline as described earlier [31]. Cross-reactive probes [29, 30], probes with SNPs with a minor allele frequency of  > 5% at the CG position or the single base extension as given by minfi; and with > 5% missing values were removed. 1872 samples and total of 697,732 probes remained for analysis.

For **LURIC,** the DNA methylation levels were quantified using the Illumina Infinium MethylationEPIC BeadChip according to the manufacturer’s protocols. In the LURIC study, quality control was implemented using the CPACOR pipeline as described earlier [32]. A total of 795,619 autosomal probes from 2423 samples were included in further analyses. CpGs located in close proximity (1–2 bp) to a genetic polymorphism in the European population with a frequency of > 0.01% as well as cross-reactive probes [33] and probes with a detection *P*-value of > 0.05 in at least 1% of the samples were removed using the rmSNPandCH function in the DMRcate package [34].

For all datasets, methylated and an unmethylated signal count per CpG site are obtained and combined into β-values, defined as the ratio of the methylated signal intensity divided by the overall signal intensity: β-value = M/(M + U + α), where an offset was added as a regularization for the situation when both M and U are low, as recommended by Illumina [35]. Untransformed beta values were utilized in all the analyses [36]. 689,101 probes overlapped with all the three discovery cohorts.

### Genome-wide genotyping in the YFS

Genomic DNA was extracted from peripheral blood leukocytes using a commercially available kit and a Qiagen BioRobot M48 Workstation according to the manufacturer’s instructions (Qiagen, Hilden, Germany). Genotyping was performed using a custom-built Illumina Human 670 k BeadChip at the Welcome Trust Sanger Institute, as described earlier [37]. Genotype imputation was performed using TOPMed r2 as reference [38].

### Genome-wide blood transcriptomics analysis in the YFS

From the 2011 follow-up of YFS (YFS 2011), whole blood was collected into PaXgene Blood RNA Tubes (PreAnalytix) and isolated with a PaXgene Blood microRNA Kit (Qiagen), including the DNase Set with the QiaCube according to the manufacturer’s instructions. The expression levels were analysed with an Illumina HumanHT-12 version 4 Expression BeadChip (Illumina Inc.) containing 47,231 expression and 770 control probes as described earlier [39]. 200 ng of RNA was reverse-transcribed into cDNA and biotin-UTP-labelled using the Illumina TotalPrep RNA Amplification Kit (Ambion); 1500 ng of cDNA was then hybridized onto the Illumina HumanHT-12 v4 Expression BeadChip. The BeadChips were scanned with the Illumina iScan system. Raw Illumina probe data were exported from GenomeStudio and analysed in R using the Bioconductor packages. The expression data were processed using non-parametric background correction, followed by quantile normalisation with control and expression probes using the *neqc* function in the *limma* package, and log2 transformation. The expression analysis was successful in 1364 samples with DNA methylation data also available.

### Liver ultrasound in the YFS

In the 2011 and 2018 follow-ups, ultrasound imaging and steatosis evaluation were performed using a validated protocol, employing Sequoia 512 ultrasound mainframes (Acuson) with 4.0 MHz adult abdominal transducers. The diagnostic evaluation of liver steatosis was performed visually using a standard system by a trained sonographer according to the following criteria: liver-to-kidney contrast, parenchymal brightness, deep beam attenuation, and bright vessel walls. Participants were further classified into those with detectable liver steatosis and those with a normal liver.

### Liver enzymes

In YFS, serum alanine aminotransferase (ALT), aspartate aminotransferase (AST), and GGT concentrations were measured with System Reagent (Beckman Coulter Biomedical). In KORA, the GGT, AST, and ALT levels were analysed according to the recommendations of the International Federation of Clinical Chemistry (IFCC) from 1983, including the optimization of substrate concentrations, the employment of NaOH, glycylglycine buffer, and sample start. Pyridoxal phosphate was applied in the assessment of ALT and AST [40]. In LURIC, GGT, ALT, AST, and alkaline phosphatase (AP) were determined using an enzymatic assay (Hitachi 717, Roche, Mannheim, Germany). Liver enzymes were measured at 25 °C. Reference values were defined as follows: GGT < 29 U/l, ALT < 23 U/l, AST < 19 U/l, AP 55–170 U/l [41]. Fatty liver index (FLI) was calculated for all the cohorts using the formula [42].

$$ FLI = \left( {e^{\begin{subarray}{l} 0.953*\log e\left( {triglycerides} \right) \\ + 0.139*BMI + 0.718*\log e\left( {GGT} \right) \\ + 0.053*waist{\kern 1pt} circumference - 15.745 \end{subarray} } } \right)/(1 + e^{\begin{subarray}{l} 0.953*\log e(triglycerides \\ + 0.139*BMI + 0.718*\log e(GGT) \\ + 0.053*waist{\kern 1pt} circumference - 15.745 \end{subarray} } )*100 $$

### Covariates

In the YFS 2011 and 2018 follow-ups, alcohol consumption data were acquired with standardized questionnaires and calculated in standard doses (12 g pure ethanol per day) by dividing the total number of doses consumed per week (0.33 L doses of beer or cider, 0.12 L doses of wine, and 0.04 L doses of hard liquor) by 7 [43]. In the YFS 1986 follow-up, participants were divided into those who drank any alcohol weekly and those who did not. For the KORA study, alcohol intake was also self-reported through a standardised interview by trained staff (g/day) [13]. In LURIC, information on alcohol consumption was collected using a questionnaire, which recorded the frequency of beer, wine and spirit consumption. The reported alcohol portions were turned into measures of volume as follows: one bottle of beer: 300 ml; one glass of wine: 100 ml; one measure of spirits: 30 ml (standard sizes that are common in Germany). The average German alcohol contents (5% for beer, 12% for wine, and 43% for spirits) were used to calculate pure alcohol consumption by multiplying the volume by the alcohol percentage and the weight of one millilitre of alcohol (0.8 g/ml) [44]. Smoking information was acquired with questionaries and categorized in YFS and KORA as smoking daily yes/no and current smokers yes/no in LURIC. In all cohorts, weight and height were measured during study visit and body mass index (BMI) calculated BMI = weight/(height*height).

## Statistical analysis

For all analyses, GGT, ALT, AST and FLI were inversetransform normalised to account for the skewed distribution of these variables. All EWAS analyses were adjusted with age, sex, BMI, smoking, alcohol consumption, estimated cell type proportions [45] and principal components of the control probes (30 for YFS and LURIC, 10 for KORA). When significant inflation was detected, BACON [46] was used to control bias and inflation in EWAS. Meta-analysis was performed using the inverse-variance-weighted method in METAL [47], combining three discovery cohorts. Individual regression models in the YFS 2018 follow-up and the longitudinal follow-up data were adjusted with age, sex, BMI, smoking, alcohol consumption, cell type proportions, and, when suitable, array type and batch as covariates in the model. Differentially methylated regions (DMRs) were identified using the *DMRcate* R package [48] as described previously [49], comparing individuals with steatotic liver to those without. FDR-corrected *p*-values (FDR < 0.05) were considered significant in EWAS and meta-analysis, but nominal p-values were reported in the replication.

To discover genetic variants, possibly affecting the association between the methylation of discovered CpG sites and liver variables, first methylation quantitative loci (meQTLs) were investigated in the GoDMC database [50]. In addition, genetic association studies were performed in YFS 2011 to identify cis-acting genetic variants (meQTLs) within 1 Mb of the CpG sites. All genetic association analyses were performed using PLINK 2.0 [51]. Statistically significant variants (*p* < 5e−8) with a minor allele frequency of ≥ 0.02 which passed the Hardy–Weinberg equilibrium check (*p* ≥ 1e−6) and had less than 5% missingness were identified. When multiple variants met these criteria, the variant with the lowest *p*-value was selected as the top-associating variant. The relationship between the discovered top variant, CpG methylation and liver phenotypes was further analysed with linear regression models predicting the liver variable, including the CpG, genetic variant, age, sex, BMI, smoking, alcohol consumption and cell type proportions in the model.

Association analysis between methylation at the identified CpGs and whole blood gene expression (Expression Quantitative Trait Methylation analysis, eQTM) was performed on gene transcripts within 100 kb of the methylation locus. Target genes were identified using the biomaRt R package [52]. Linear regression analyses predicting transcript expression were adjusted for age, sex, BMI, smoking and alcohol consumption. Mediation analysis was performed if the CpG and a nearby gene, whose expression correlated with the methylation levels (nominal *p* < 0.05), both associated with the liver variable in question.

## Mediation analysis

For each CpG methylation–gene expression pair identified according to the criteria above, a separate mediation analysis was performed to investigate whether the effect of DNA methylation on FLI was mediated through the expression levels of the nearby gene [53]. The assumed causal associations of the mediation question as two directed acyclic graphs are presented in Supplementary Fig. 1. In observational studies, certain assumptions pertaining to confounding are required. Confounding variables (confounders) are the common causes of DNA methylation, gene expression and/or FLI. These so-called exchangeability conditions are:

1. No unmeasured confounding of exposure-outcome (DNAm-FLI) relationship
2. No unmeasured confounding of mediator-outcome (GE-FLI) relationship
3. No unmeasured confounding of exposure-mediator (DNAm-GE) relationship
4. No mediator-outcome confounders that are affected by the exposure; i.e., DNA methylation cannot affect any common cause of gene expression and fatty liver index.

If these conditions are fulfilled, the individuals with certain levels of exposure and mediator can be considered as “exchangeable” conditionally on the confounders, i.e., the exposure and mediator can be treated as if they were randomized and confounding can be ruled out as the explanation of any associations observed. Thus, the mediation effects of interest delineated below can be identified based on observational data [54].

Under the exchangeability conditions above, the mediation effects came be obtained from the observed data. In practice, this was accomplished by fitting the following three linear regression models:

1 $$ FLI = \eta_{0} + \eta_{1} DNAm + \user2{ \eta }_{2}^{\user2{^{\prime}}} {\boldsymbol{C}} $$

2 $$ FLI = \gamma_{0} + \gamma_{1} DNAm + \gamma_{2} GE + {\boldsymbol{\gamma}}_{{\mathbf{3}}}^{\user2{^{\prime}}} {\boldsymbol{C}} $$

3 $$ GE = \beta_{0} + \beta_{1} DNAm + {\boldsymbol{\beta}}_{{\mathbf{2}}}^{\user2{^{\prime}}} \user2{C }, $$

where GE denotes the gene expression level, DNAm the DNA methylation, and C includes all covariates adjusted for in each model: age, sex, smoking and alcohol use categorized into 0, 0–1 or more than 1 drink per day. Parameter ${\eta }_{1}$ descibes the total effect, i.e., the effect of DNA methylation on (normalized) FLI when not accounting for gene expression, while ${\gamma }_{1}$ corresponds to the direct effect, the effect of DNA methylation on FLI when taking gene expression into account (pathway (a) in Supplementary Fig. 1A). The indirect effect of DNA methylation on FLI via gene expression was calculated utilising the product-of-coefficients approach by multiplying the parameters ${\beta }_{1}$ and ${\gamma }_{2}$ (pathways (c) and (d) in Supplementary Fig. 1B), and its standard error was obtained using the multivariate Delta method [55]. The proportion mediated was calculated by dividing the indirect effect with the total effect, and its standard error was also obtained with the multivariate Delta method. Mediation was inferred if the 95% confidence interval of the indirect effect did not include zero. The sample was stratified into normal-weight participants (BMI < 25 kg/m^2^, n = 524) and overweight/obese participants (BMI $\ge \hspace{0.17em}$25 kg/m^2^, n = 686) and mediation analyses were conducted separately for both strata.

## Results

### Methylation levels of cg06690548 (*SLC7A11)* are associated with presence of liver steatosis

**Fig. 2:**
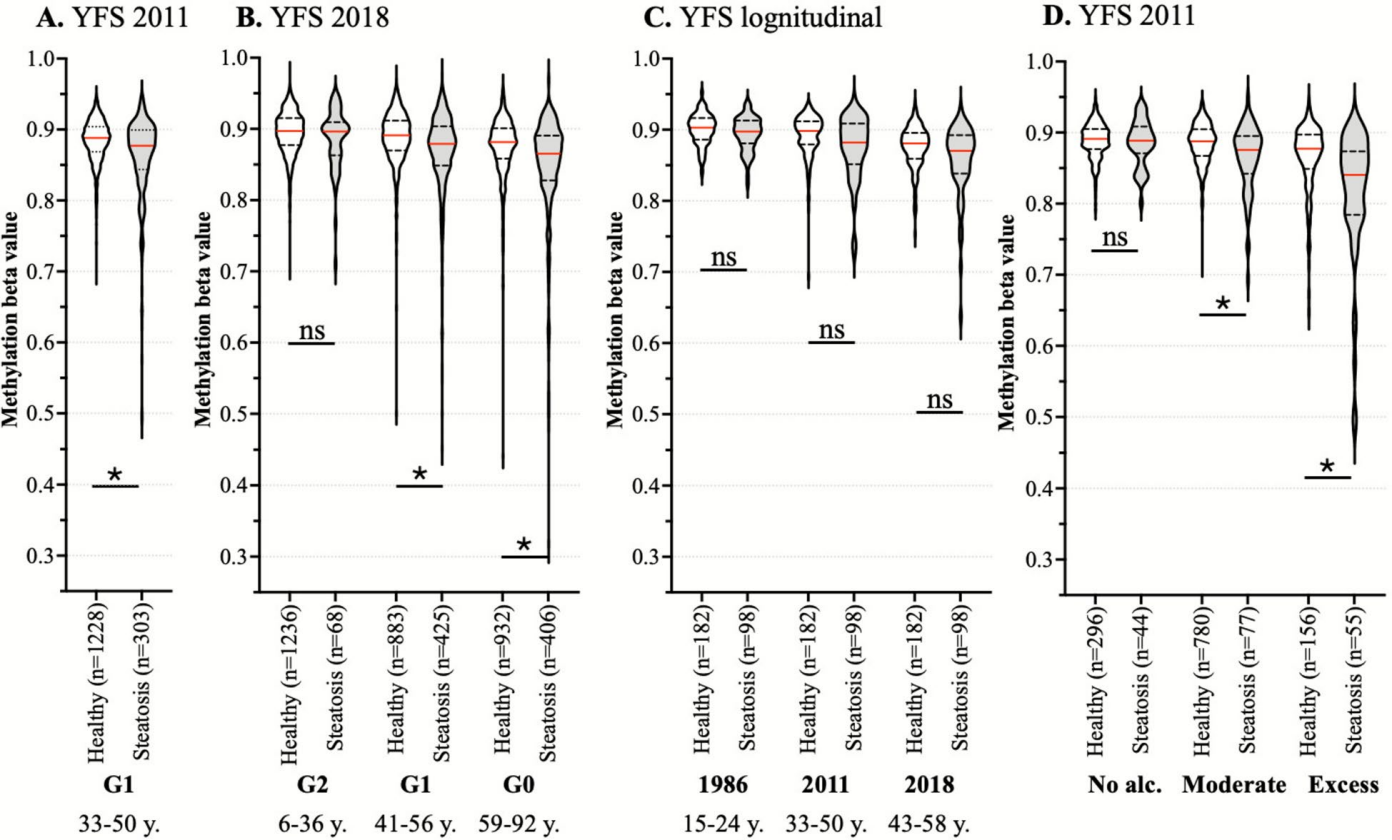
Methylation of cg06690548 (*SLC7A11*) in the YFS **A** 2011 follow-up, where cg06690548 was the only CpG which was statistically (FDR < 0.05) differently methylated in individuals with and without liver steatosis; **B** 2018 follow-up in the follow-up cohort (G1) and their children (G2) and parents (G0). The difference in methylation levels of cg06690548 (*SLC7A11*) between those with and without liver steatosis was statistically significant (*p* < 0.05) in G1 and G0, but not in the younger G2; **C** longitudinal YFS data from G1 from the 1986, 2011 and 2018 follow-ups. In the smaller subset of individuals with methylation data available from three timepoints (n = 255), methylation levels of cg06690548 (*SLC7A11*) were already lower in 1986 in individuals who develop MASLD by 2018. Methylation levels of this site decrease in the whole population and the median difference between those who do and do not develop liver steatosis by 2011 slightly increases when the individuals age. After adjusting with age, sex, BMI, smoking, alcohol consumption and estimated cell proportions (as all models), the differences between those who do or do not develop liver steatosis by 2018 were not statistically significant in any timepoint; **D** YFS 2011 follow-up stratified with alcohol consumption. Association between cg06690548 (*SLC7A11*) methylation and presence of liver steatosis was non-significant in individuals who reported to consume no alcohol at all and greatest in the subpopulation who reported excess alcohol consumption. *—*p* < 0.05; ns—*p* > 0.05

In YFS 2011 (33–50 years, n = 1529), we identified that only the methylation levels of cg06690548 in the body of *SLC7A11* were significantly (FDR < 0.05) different between those with and without liver steatosis (nominal *p* = 5.55 × 10^–9^, FDR = 0.004, regression beta = − 0.018, delta methylation beta = − 0.011, Fig. 2A). This difference in cg06690548 (*SLC7A11)* methylation levels was also observed after a 7- to 9-year follow-up of the same cohort (YFS 2018 G1, age 41–56 years, nominal *p* = 0.018, regression beta = − 0.008, delta methylation beta = − 0.012), and a similar methylation pattern was also observed in the parents of the original cohort (YFS 2018 G0, age 59–92 years, nominal *p* = 4.18 × 10^–13^, regression beta = − 0.016, delta methylation beta = − 0.016) (Fig. 1B). Levels of cg06690548 (*SLC7A11)* methylation were also slightly lower in individuals with liver steatosis in the children of the original follow-up cohort, but the association was not statistically significant (YFS 2018 G2, age 6–36 years, nominal *p* = 0.277, regression beta = − 0.005, delta methylation beta = − 0.001).

We then analysed the methylation level of cg06690548 (*SLC7A11)* in a longitudinal setting in a subset of 255 individuals from YFS with DNA methylation data available from 1986, 2011, and 2018. Those who developed liver steatosis by 2018 (n = 98) had lower levels of cg06690548 (*SLC7A11)* methylation across all three timepoints (delta methylation beta 1986 = − 0.006, 2011 = − 0.016 and 2018 = − 0.011), but the difference was not statistically significant (*p*> 0.05) in this smaller population subset at any of the timepoints (Fig. 2C). The association between cg06690548 methylation and the presence of liver steatosis remained significant in the YFS 2011 follow-up when investigating individuals who consumed alcohol moderately (≤ 350 g/week for females and ≤ 420 g/week for males^1^) or not at all (nominal *p* = 6.37 × 10^–4^, regression beta = − 0.004, delta methylation beta − 0.020, n = 1269). However, the association was not significant in individuals who reported not drinking alcohol (nominal *p* = 0.501, regression beta = − 0.011, delta methylation beta = − 0.003 n = 333). The greatest difference between individuals with and without steatosis was seen in those who consumed alcohol excessively (nominal *p* = 0.008, regression beta = − 0.036, delta methylation beta = − 0.051, n = 131) (Fig. 2D).

DMR analysis revealed one DMR (chr2:10,184,444–10184650) overlapping the transcription factor *KLF11*, with three CpGs that were hypomethylated in individuals with liver steatosis in comparison to those without (FDR = 1.63 × 10^–9^, max difference − 0.014, mean difference − 0.009).

### Meta-analysis reveals systematic associations between blood DNA methylation and GGT levels and FLI

We identified CpGs associated with liver enzyme levels in three discovery cohorts (YFS 2011, KORA and LURIC) and aimed to replicate these associations again in the three-generational YFS 2018 follow-up to assess their potential longitudinal change and the effect of the age of the cohort on the discovered associations. Only cg00078197 (in the body of *HGH1* homolog) and cg14141451 (in the body of scribble planar cell polarity protein *SCRIB*) were associated with ALT levels in the YFS 2011 follow-up (nominal *p* = 1.09 × 10^–8^, FDR = 0.008 regression beta = 0.003 and nominal *p* = 5.75 × 10^–8^, FDR = 0.022 regression beta = 0.018). No significant associations were discovered for ALT in KORA or LURIC. Similarly, no CpG sites were associated with AST levels in any of the discovery cohorts.

**Fig. 3:**
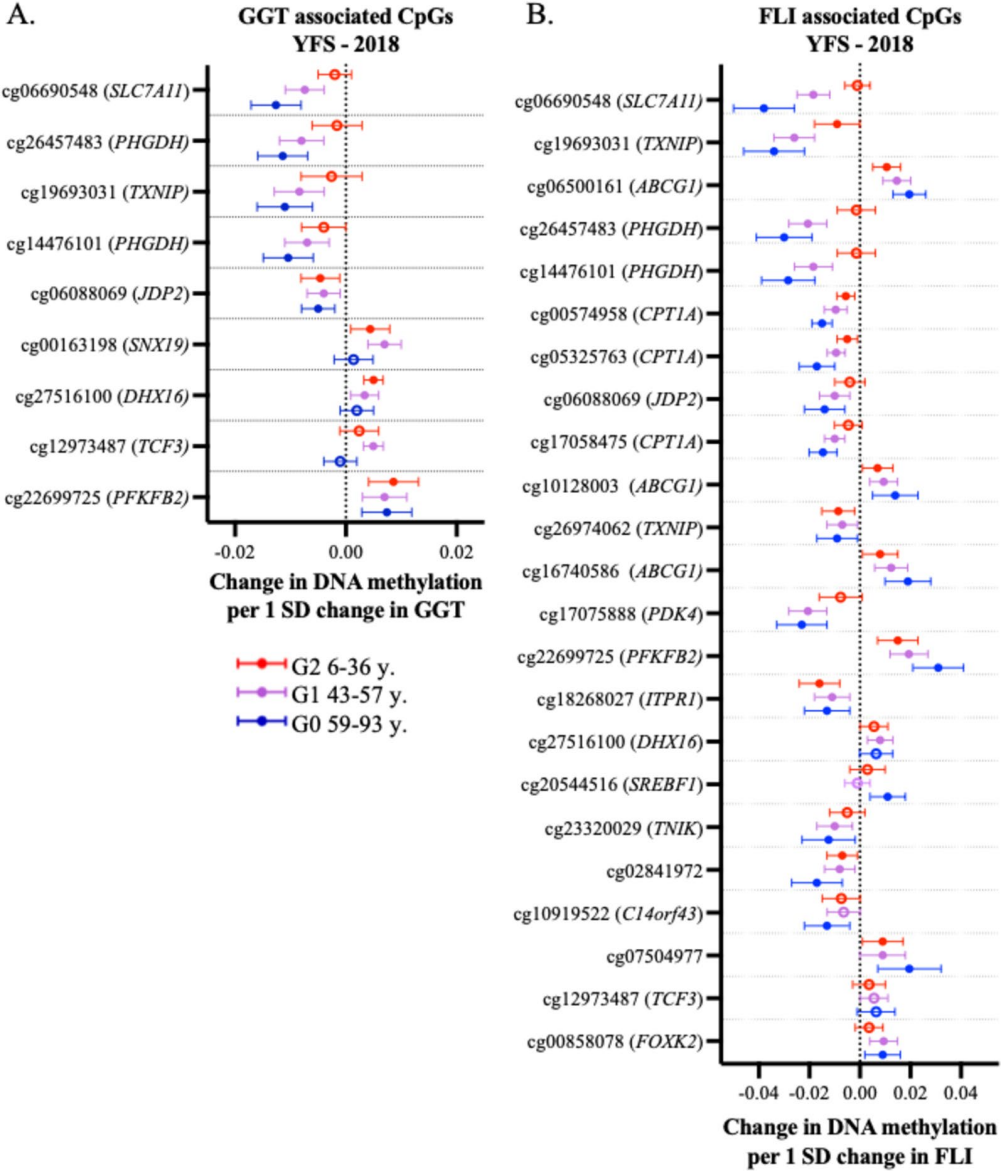
The associations between DNA methylation levels in CpGs identified and **A** GGT and **B** FLI in the three generational YFS 2018 follow-up. Many of the CpGs show stronger associations with the given liver variable in the older G0 (59–93y.) and G1 (43–58y.) populations. Intriguingly, for example, the methylation of cg27516100 (*DHX16*) associates statistically significantly with GGT or FLI only in the young G2 and G1, and not in the G0 population. All models are adjusted with age, sex, BMI, smoking, alcohol consumption and estimated cell type proportions. *NOTE* X-axis of the **A** and **B** figure are not on the same scale

For GGT EWAS results, we identified significant inflation in LURIC (λ = 1.47) which was decreased but not fully controlled by the BACON correction (λ = 1.08) and thus we selected only those CpG sites that were found to be FDR-significantly associated with GGT levels in at least two cohorts for further investigation (Table 1). In the three-generational YFS 2018 follow-up, we observed that the association between cg06690548 (*SLC7A11*), cg26457483 (*PHGDH*)*,* cg19693031 *(TXNIP*), and cg14476101 (*PHGDH*) and GGT levels were stronger in the older generations compared to the younger generations. In contrast, the association between cg06088069 (*JDP2*) and cg22699725 (*PFKFB2*) was equally strong in all generations. Interestingly, the association between cg00163198 (*SNX19*), cg27516100 (*DHX16*) and GGT levels was significant in G1 and G2, but not in the oldest G0 population (Fig. 3A).

**Table 1:** The association between GGT levels and whole blood DNA methylation in the discovery analysis

IlmnID	Gene	YFS (n = 1526)				KORA FF4 (n = 1872)				LURIC (n = 2371)				Meta-analysis (n = 5769)			
		Beta	SE	*P*-value	FDR	Beta	SE	*P*-value	FDR	Beta	SE	*P*-value	FDR	Zscore	*P*-value	FDR	Direction
cg06690548	*SLC7A11*	− 0.006	0.001	1.18E–07	0.014	− 0.008	0.001	1.93E–13	1.34E–07	− 0.014	0.001	1.42E–17	1.09E–11	− 12.383	3.22E–35	2.50E–29	−−−
cg26457483	*PHGDH*	− 0.008	0.002	5.02E–08	0.009	− 0.007	0.001	2.30E–07	0.023	− 0.014	0.001	3.93E–15	1.52E–09	− 10.787	3.96E–27	1.54E–21	−−−
cg19693031	*TXNIP*	− 0.009	0.002	1.31E–07	0.014	− 0.009	0.001	3.31E–10	1.16E–04	− 0.010	0.001	9.51E–11	7.34E–06	− 10.444	1.56E–25	4.05E–20	−−−
cg14476101	*PHGDH*	− 0.009	0.002	5.79E–08	0.009	− 0.008	0.002	1.11E–07	0.015	− 0.012	0.001	1.65E–12	1.82E–07	− 10.341	4.61E–25	8.97E–20	−−−
cg06088069	*JDP2*	− 0.003	0.001	3.12E–05	0.265	− 0.004	0.001	1.56E–07	0.018	− 0.006	0.001	8.53E–15	2.19E–09	− 10.104	5.32E–24	8.28E–19	−−−
cg00163198	*SNX19*	0.006	0.001	1.98E–08	0.005	0.004	0.001	9.91E–07	0.052	0.005	0.001	5.22E–09	1.35E–04	9.419	4.54E–21	5.89E–16	+++
cg27516100	*DHX16*	0.003	0.001	0.001	0.457	0.004	0.001	3.28E–09	0.001	0.005	0.001	1.88E–09	7.58E–05	8.891	6.07E–19	5.91E–14	+++
cg12973487	*TCF3*	0.002	0.001	0.047	0.741	0.004	0.001	4.65E–07	0.032	0.004	0.001	3.47E–07	0.002	7.16	8.09E–13	3.00E–08	+++
cg22699725	*PFKFB2*	0.007	0.001	2.21E–09	0.002	0.005	0.001	4.25E–07	0.032	0.000	0.001	0.993	1.000	5.953	2.63E–09	2.12E–05	++−

Only CpGs associating with GGT significantly (FDR < 0.05) in at least 2 of the cohorts are presented. All analyses are adjusted with age, sex, BMI, smoking, alcohol consumption, estimated cell proportions and technical principal components (PCs). Manhattan plot of the meta-analysis results in Supplementary Fig. 2A

We further investigated the association between FLI and blood DNA methylation levels in the three discovery cohorts. Similarly, with GGT, LURIC results presented inflation (λ = 1.41), which decreased by BACON (λ = 1.07). After the correction, we discovered 23 sites that were significantly associated with FLI in at least two cohorts. Among them, eight CpGs were also associated with GGT levels in at least two discovery cohorts (Table 2). In addition, this analysis revealed associations between FLI and the methylation in *ABCG1* (3 CpGs) and *CPT1A* (3 CpGs) (Table 2). When analysing the associations between these 23 CpGs and FLI in YFS 2018 follow-up, CpGs locating near or at *TXNIP, SLC7A11, PHGDH, JDP2, PDK4, TNIK* had stronger negative association in older generations, while a similar but positive pattern could be seen in CpGs locating near or at *ABCG1, PHGDH* (Fig. 3B).

**Table 2:** The association between fatty liver index (FLI) and whole blood DNA methylation in the discovery analysis

IlmnID	Gene	YFS (n = 1526)				KORA FF4 (n = 1872)				LURIC (n = 2371)				Meta-analysis (n = 5769)			
		Beta	SE	*P*-value	FDR	Beta	SE	*P*-value	FDR	Beta	SE	*P*-value	FDR	Zscore	*P*-value	FDR	Direction
cg06690548	*SLC7A11*	− 0.014	0.002	2.16E–09	2.38E–04	− 0.008	0.001	1.93E–13	1.34E–07	− 0.026	0.002	2.33E–22	8.99E–17	− 14.160	1.63E–45	1.27E–39	−−−
cg19693031	*TXNIP*	− 0.02	0.003	2.71E–10	5.22E–05	− 0.009	0.001	3.31E–10	1.16E–04	− 0.022	0.002	6.93E–19	1.07E–13	− 14.002	1.52E–44	4.33E–39	−−−
cg06500161	*ABCG1*	0.012	0.002	9.25E–12	2.49E–06	0.001	0.001	0.08	0.828	0.013	0.001	2.17E–19	4.19E–14	13.995	1.67E–44	4.33E–39	+++
cg26457483	*PHGDH*	− 0.016	0.003	4.85E–08	0.004	− 0.007	0.001	2.30E–07	0.023	− 0.028	0.002	3.34E–24	2.57E–18	− 13.826	1.77E–43	3.44E–38	−−−
cg14476101	*PHGDH*	− 0.017	0.003	8.57E–08	0.005	− 0.008	0.002	1.11E–07	0.015	− 0.025	0.002	8.70E–22	2.24E–16	− 13.571	5.99E–42	9.32E–37	−−−
cg00574958	*CPT1A*	− 0.008	0.001	2.44E–13	1.88E–07	− 0.001	0	0.022	0.74	− 0.005	0.001	2.18E–16	1.68E–11	− 13.032	8.03E–39	1.04E–33	−−−
cg05325763	*CPT1A*	− 0.009	0.002	7.87E–10	1.21E–04	− 0.002	0.001	0.008	0.664	− 0.008	0.001	9.41E–19	1.21E–13	− 12.797	1.72E–37	1.91E–32	−−−
cg06088069	*JDP2*	− 0.006	0.001	5.17E–05	0.216	− 0.004	0.001	1.56E–07	0.018	− 0.010	0.001	2.23E–18	2.46E–13	− 11.264	1.98E–29	1.92E–24	−−−
cg17058475	*CPT1A*	− 0.007	0.002	2.04E–06	0.048	− 0.002	0.001	0.005	0.619	− 0.006	0.001	3.01E–14	1.55E–09	− 11.082	1.53E–28	1.19E–23	−−−
cg10128003	*ABCG1*	0.01	0.002	8.76E–08	0.005	0.001	0.001	0.414	0.946	0.008	0.001	3.87E–14	1.86E–09	10.448	1.50E–25	8.36E–21	+++
cg26974062	*TXNIP*	− 0.008	0.002	1.86E–05	0.142	− 0.003	0.001	8.98E–08	0.015	− 0.005	0.001	2.48E–06	0.008	− 9.688	3.40E–22	1.65E–17	−−−
cg16740586	*ABCG1*	0.01	0.002	5.44E–07	0.02	0.001	0.001	0.3	0.922	0.012	0.002	4.76E–12	1.93E–07	9.654	4.71E–22	2.16E–17	+++
cg17075888	*PDK4*	− 0.016	0.003	2.68E–07	0.012	− 0.005	0.001	4.17E–05	0.247	− 0.011	0.002	1.50E–08	2.23E–04	− 9.616	6.83E–22	2.95E–17	−−−
cg22699725	*PFKFB2*	0.016	0.002	9.72E–12	2.49E–06	0.005	0.001	4.25E–07	0.032	0.003	0.002	0.058	0.744	9.248	2.28E–20	7.71E–16	+++
cg18268027	*ITPR1*	− 0.011	0.002	1.97E–06	0.047	− 0.004	0.001	4.28E–06	0.116	− 0.006	0.001	2.45E–10	6.14E–06	− 9.110	8.22E–20	2.46E–15	−−−
cg27516100	*DHX16*	0.005	0.002	0.003	0.516	0.004	0.001	3.28E–09	0.001	0.008	0.001	1.06E–11	3.72E–07	8.830	1.04E–18	3.01E–14	+++
cg20544516	*SREBF1*	0.007	0.001	9.03E–07	0.027	0.001	0.001	0.108	0.849	0.008	0.001	1.06E–11	3.72E–07	8.762	1.91E–18	5.32E–14	+++
cg23320029	*TNIK*	− 0.013	0.002	1.12E–07	0.006	− 0.003	0.001	0.002	0.583	− 0.007	0.001	1.92E–08	2.55E–04	− 8.580	9.51E–18	2.55E–13	−−−
cg02841972		− 0.01	0.002	1.21E–06	0.033	− 0.004	0.001	6.29E–04	0.493	− 0.009	0.001	3.17E–08	3.65E–04	− 8.537	1.38E–17	3.58E–13	−−−
cg10919522	*C14orf43*	− 0.011	0.002	6.68E–08	0.005	− 0.002	0.001	0.014	0.707	− 0.008	0.001	1.31E–07	0.001	− 8.412	4.03E–17	9.79E–13	−−−
cg07504977		0.012	0.002	1.68E–06	0.043	0.002	0.001	0.082	0.83	0.010	0.002	5.03E–06	0.012	7.845	4.34E–15	7.67E–11	+++
cg12973487	*TCF3*	0.004	0.002	0.044	0.726	0.004	0.001	4.65E–07	0.032	0.008	0.001	6.79E–08	0.001	6.968	3.21E–12	3.09E–08	+++
cg00858078	*FOXK2*	0.011	0.002	1.07E–07	0.006	0.002	0.001	0.034	0.771	0.005	0.001	1.74E–05	0.026	6.803	1.03E–11	8.59E–08	+++

Only CpGs associating with FLI significantly (FDR < 0.05) in at least 2 of the cohorts are presented. All analyses are adjusted with age, sex, BMI, smoking, alcohol consumption, estimated cell proportions and technical PCs. Manhattan plot of the meta-analysis results in Supplementary Fig. 2B

In a longitudinal setting, we analysed whether youth methylation levels (G1 1986 follow-up) of the CpGs associating with liver health variables cross-sectionally predicted 2018 GGT or FLI levels (n = 255 for those CpGs that are in 450 K and EPIC array, n = 98 for those CpGs that are only in EPIC array) No statistically significant association were found (*p* > 0.1 for all analysis).

### Association between the CpG sites and GGT or FLI is affected by meQTLs

We first identified meQTLs using the GoDMC database, based on genetic and methylation data from over 32,000 individuals. Of the CpG sites associated with GGT, only cg19693031 (*TXNIP*) and cg14476101 (*PHGDH*) had genome-wide level meQTLs in GoDMC. This was expected, as only three of the 9 identified CpGs are present in 450 K array used in the GoDMC analyses. We identified 3 of the 9 CpG sites associated with GGT as having meQTLs in YFS (Supplementary Table 2). To analyse whether the meQTLs were the cause of the association between the CpG methylation and GGT levels, we included the most significant SNP as a cofactor in the model predicting GGT levels in YFS 2011 follow-up data. The inclusion of the SNP did not modify the associations between the CpGs and GGT levels, and none of the meQTLs discovered in YFS had independent association with GGT levels (nominal *p* > 0.05 for all) (Supplementary Table 2).

Of the 23 CpGs that were associated with FLI in the discovery cohorts, six and nine had a cis-meQTLs in the GoDMC database and in the YFS data, respectively. Six of the meQTLs discovered in the YFS data did not overlap with the CpGs and SNPs discovered for GGT. As with the CpGs and SNPs associated with GGT, all the CpGs that associated with FLI in YFS in the initial analysis also remained significantly associated after adjusting with the most significant meQTL (Supplementary Table 3). None of the discovered meQTLs associated with FLI levels in the YFS (nominal *p*  > 0.05 for all).

### Association between cg06500161 (*ABCG1*) and cg20544516 (*SREBF*) methylation and FLI was mediated by the expression of nearby genes

In YFS, the methylation levels of all CpGs associated with GGT, except cg06690548 (*SLC7A11*) and cg00163198 (*SNX19*), were significantly associated with the whole blood expression of genes located within 100 kb of the methylation locus. Strong negative associations were seen especially between cg27516100 (*DHX16*) and the expression of IER3, KIAA1949, FLOT1 and PPP1R10; cg26457483 and cg14476101 (*PHGDH*) and PHGDH expression; cg06088069 (*JDP2*) and JDP2 expression; cg22699725 (*PFKFB2*) and PFKFB2 expression. Positive associations were observed between cg27516100 (*DHX16*) and the expression of MDC1, TUBB, ABCF1, GNL1, MRPS18B and DHX16; cg12973487 (*TCF3*) and the expression of MBD3 and TCF3. Of the analysed transcripts expression of ZNF697, GNLI and MRPS18B associated with GGT levels with nominal significance (Supplementary Table 4).

CpG sites associated with FLI in the proximity of *ABCG1* (cg06500161, cg10128003, cg16740586) and *CPT1A* (cg00574958, cg05325763 and cg17058475) negatively associated with the expression of multiple transcripts of the corresponding gene. Similarly, cg20544516 (*SREBF*) negatively associated with two distinct transcripts from this gene and cg02841972 with the expression of three *KLF11* transcripts. Further negative associations were seen between cg17075888 (*PDK4*) and PDK4 expression; cg07504977 and SCD expression; and cg23320029 (*TNIK*) and TNIK expression (Supplementary Table 5). The expression of several of these transcripts also independently associated with FLI levels (Supplementary Table 5).

**Fig. 4:**
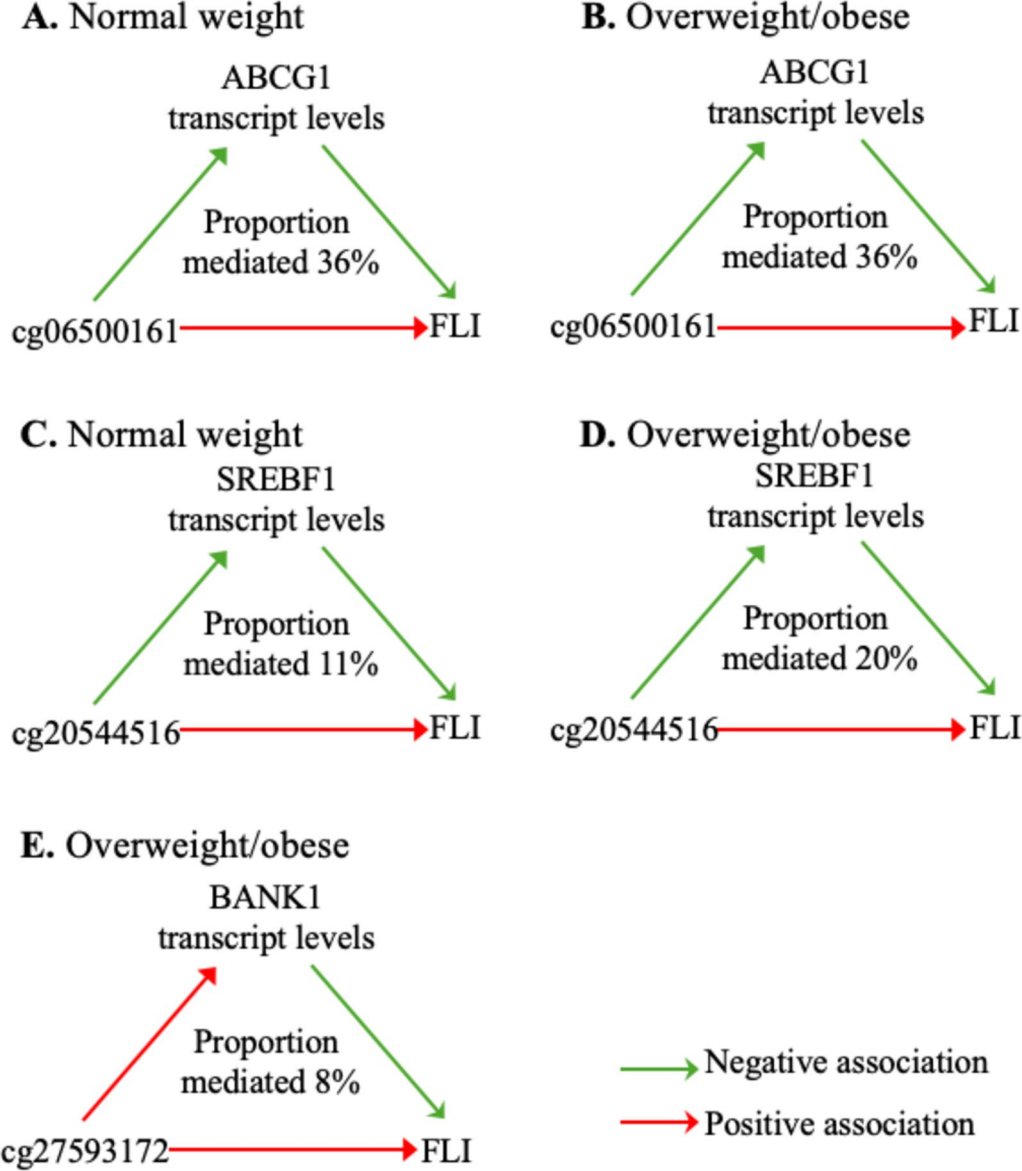
Nearby transcripts mediating the effect of CpG methylation on FLI in the YFS 2011 follow-up. Methylation levels of cg06500161 (*ABCG1*) and cg20544516 (*SREBF1*) on FLI were mediated by the blood expression of nearby transcripts in both the normal-weight (n = 542) and overweight/obese (n = 686) subpopulations, while mediation effect of cg27593172 (**E**) on BANK1 expression could only be seen in the overweight/obese subpopulation. In E, the direction of direct effect and indirect effect are different, indicating a more complex association than direct regulation of gene expression by methylation

We further investigated whether the blood gene expression of the nearby transcripts could mediate the association between CpGs and FLI in the YFS 2011 population. In both normal-weight and overweight/obese subpopulations, mediation by the expression of nearby transcripts was observed for cg06500161 (*ABCG1*) (proportion mediated: 36% for both strata) and cg20544516 (*SREBF*) (proportion mediated: 11% and 20% for normal-weight and overweight/obese, respectively). In addition, a mediation effect was indicated only in the overweight/obese subpopulation with cg27593172 and BANK1 transcript, however, the directions of direct and indirect effect were different, which could reflect an alternative mechanism rather than gene expression regulation by DNA methylation (Supplementary Table 6, Fig. 4).

## Discussion

The presence of ultrasonically-identified steatotic liver associated only with the methylation levels of cg06690548 in *SLC7A11.* This finding was replicated two of the oldest generations (G1 and G0) but not in the youngest generation (G2) of the three-generational YFS 2018 follow-up. Meta-analysis revealed highly replicative findings between blood DNA methylations levels and GGT and FLI. Most of these associations were stronger in the older generations of the YFS 2018 follow-up, while only a few associated with liver variables in the youngest participating generation (age 6–36 years). Although we found several meQTLs associating with these CpGs, the association between the methylation of the CpGs and the liver variables was independent of genetic variation. We further report associations between the discovered CpGs and whole blood gene expression of nearby genes, indicating a possible biological function of the methylation changes.

Methylation at cg06690548 in *SLC7A11* was the only CpG site associated with the presence of liver steatosis in YFS 2011. The methylation of this site was also strongly associated with GGT levels and FLI in the meta-analysis. Previously, methylation at this CpG has been associated with liver steatosis [18], NAFLD [19], and the levels of GGT and ALT [18] in mostly European populations. However, it did not associate with liver fat content in a Chinese population [20]. We observed that methylation levels of this CpG were already lower in individuals in their twenties who later developed liver steatosis by their fifties; however, in general, the association of this CpG and liver variables was stronger in the older population (G0). On the other hand, Melton et al. did not observe an association between cg06690548 methylation and steatosis score or NAFLD in adolescence (mean age 17 years) [56]. Although Nano et al. suggest that methylation in *SLC7A11* could be causal for elevated GGT levels, our results indicate that the changes in methylation of this site are accompanied by the worsening metabolic health. In line with this hypothesis, methylation of cg06690548 has been previously associated with BMI [57–60], CRP, glucose [57, 61] and insulin levels [57, 61], T2D [62], triglyceride levels [57] and alcohol consumption [63–66]. Even though we adjusted all our analyses with age, BMI and self-reported alcohol consumption, we cannot rule out that these confounding factors could be causal for the observed changes in methylation at this site, rather than the liver condition itself. The association between liver steatosis and cg06690548 methylation levels was observed in the subpopulation of YFS who were modest alcohol consumers, but not in those who reported no alcohol consumption. These results do not rule out the possibility that the association between cg06690548 methylation and liver steatosis is influenced by alcohol consumption; however, due to the small number of individuals in analysis (n = 333) and especially the number with liver steatosis (n = 44), the lack of association in the teetotalers could also be due to limited study power.

*SLC7A11* is an amino acid transporter of cysteine and glutamate and it has a well-established role in protecting cells from ferroptosis [67]. It has been implied to counteract effects of oxidative stress and maintain triglyceride metabolism in primary hepatocytes [68]. Knocking down SLC7A11 from liver cells lead to a significant decrease in lipid-associated genes and alterations in lipid metabolism, indicating that *SLC7A11* has a role in maintaining lipid homeostasis in the liver [18]. Even though we, or Ma et al. [19], could not identify an association between cg06690548 and SLC7A11 expression in blood, Vallegra et al. [69] report an association utilising summary-based mendelian randomisation (SMR) analysis [70]. Negative correlation has been observed between *SLC7A11* methylation and gene expression in hepatocellular carcinoma [71] and Simner et al. demonstrated that decreasing methylation via 5-AZA-dC treatment in trophoblast-derived BeWo cells increased SLC7A11 levels [72], both indicating that DNA methylation could regulate SLCA11 expression.

We discovered one DMR between individuals with and without liver steatosis in YFS 2011. Although none of the CpGs within the DMR chr2:10184444–10184650 were individually statistically significantly associated with the presence of liver steatosis or other liver health indicators investigated here, methylation of nearby cg02841972 (chr2:10176151) associated with FLI levels and with the expression of KLF transcription factor 11 (KLF11), overlapping the DMR (chr2: 10183677–10194963). KLF11 is a transcription factor that regulates glucose metabolism and insulin secretion. Rare mutations in KLF11 are thought to be causal for maturity-onset diabetes of the young type 7, while a more common variant has been associated with T2D in northern European populations [73]. Methylation levels in the body of *KLF11,* previously associated with insulin resistance (cg20853880 and cg05301188) [74], and cg02841972 upstream of *KLF11*, associated here with FLI, have previously been associated with T2D [62] and CRP [75], further linking methylation in this region with the metabolic dysfunction underlying the co-morbidity of T2D and MASLD.

In addition to cg06690548 (*SLC7A11)*, out of the 9 CpG sites which associated with GGT levels in at least two of our discovery cohorts, blood methylation levels of cg19693031 (*TXNIP*) and cg14476101 (*PHGDH*) have been previously associated with NAFLD in adults [19], with cg14476101 (*PHGDH*) also associating with steatosis score and NAFLD in adolescence [56]. Methylation of cg00163198 (*SNX19*), measured only with the EPIC array (not 450 K), has also previously been associated with liver fat content [20].The lack of replication is partly due to the fact that only three of the discovered sites are present in Illumina 450 K array, previously used in the majority of analyses. In the three-generational YFS 2018 data, cg06690548 (*SLC7A11*), cg14476101 and cg26457483 (*PHGDH*), and cg19693031 (*TXNIP*) showed significant association with GGT only in the older G1 (43–58 years) and G0 (59–93 years) generations, with the strongest associations in G0. The difference is intriguing, as G1 and G0 have very similar BMIs, liver enzyme levels and prevalence of liver steatosis, and G0 had consumed less alcohol than G1. As G0 are the parents of G1, genetic differences should not offer a major explanation either. Of these, the negative association of cg19693031 (*TXNIP*) with liver fat content in longitudinal analysis has also been previously reported [20]. Even more perplexing is the association between cg27516100 (*DHX16*) and cg00163198 (*SNX19*) with GGT levels in the younger G2 and G1, but not in G0.

Even though meQTLs were discovered for CpGs in *PHGDH* (cg14476101 and cg26457483) and cg22699725 (*PFKFB2*), none associated with GGT levels or attenuated the association between the methylation sites and GGT. Similarly, although most of the CpGs associated with GGT also associate with the expression of nearby genes, the levels of these transcripts were not strongly associated with GGT levels. It must be noted that our gene expression data are from peripheral blood and, for example, changes in PHGDH expression in the liver have been linked to lipid-associated genes, indicating a role in liver function [76].

In addition to those CpGs associating with GGT, we discovered 15 additional CpGs associating with FLI in at least two of our discovery cohorts. Of these, cg06500161 (*ABCG1*) has been associated with NAFLD [19] and liver fat content [20] in a cross-sectional setting, and cg06500161 and cg16740586 (*ABCG1*) also in longitudinal analysis [20], while cg00574958 (*CPT1A*) has been associated with NAFLD in a cross-sectional setting [19]. ABCG1 facilitates cholesterol efflux to HDL, whereas CPT1A catalyses the rate-limiting step of beta-oxidation of long-chain fatty acids. Methylation of these genes has been strongly linked to obesity [57], and in previous analyses reporting their association with NAFLD and liver fat content, these associations diminished after adjusting with BMI [19, 20]. Methylation levels in *SREBF1*, a transcription factor regulating de novo lipogenesis, have been associated with liver steatosis in many studies [18–20, 56]. However, while these studies report an association between cg11024682 (*SREBF1*) and liver steatosis, we observe an association between cg20544516 (*SREBF1*) and FLI. Instead of liver steatosis, cg20544516 has previously been associated with T2D [62] and triglyceride levels [77], linking it closely to the development of MASLD.

In the three generations participating the YFS 2018 follow-up, most of the DNA methylation sites associating with FLI show the strongest association in the oldest G0 and the weakest association in the youngest G2 generation. Interestingly, cg22699725 (*PFKFB2*) and cg06088069 (*JDP2*), which had similar association levels with GGT in all generations, have an increasing association with FLI from the youngest to the older generations. This highlights that these indicators of liver health could be describing different aspects of the underlying pathology.

We also identified several meQTLs for CpGs associated with FLI. Similar to those associated with GGT, adding meQTL in the model predicting FLI levels did not diminish the association—and in some cases, such as cg14476101 (*PHGDH1*), cg17075888 (*PDK4*) and cg10919522 in *C14orf43*, adding genetic variation to the model even strengthened the association between the CpG and FLI. Unlike in the GGT analysis, transcripts associated with FLI-linked CpGs were often themselves associated with FLI. For example, the effects of cg06500161 (*ABCG1*) and cg20544516 (*SREBF*) methylation on FLI levels were mediated by the blood transcript levels of these genes. This indicates that the link between these CpG sites and FLI could be more systemic—rather than liver-specific—and that the biological link between methylation and FLI levels may, at least partly, involve regulation of nearby gene expression.

Like all studies, also ours has some limitations. Firstly, the YFS longitudinal data encompassing the 1986, 2011, and 2018 follow-ups is limited in size and consists of several batches. Therefore, we chose not to perform true longitudinal analysis but instead conducted repeated cross-sectional analyses. Liver enzyme levels were also not measured in the YFS 1986 follow-up, further limiting longitudinal analyses. The datasets utilised here consist of mainly white Europeans, and the results cannot be directly generalised to other ethnicities. Importantly, we observed some discrepancies between the Finnish datasets and the German, cg12973487 (*TCF3*) which associated significantly with FLI in German cohorts but not in any of the populations or settings in the Finnish YFS. From all the cohorts, the DNA methylation levels have been measured from blood leukocytes, and the associations should thus be considered to reflect the systematic components of liver steatosis and the crosstalk between the liver and the immune system. In the mediation analysis, even though the confounders deemed relevant in the mediation question were controlled for, due to the cross-sectional nature of these analyses, reverse causality cannot be fully ruled out as an explanation of the results. Thus, rather than interpreting these results through a strictly causal lens, the mediation analysis should be interpreted as a statistical decomposition of the association between DNA methylation and FLI.

Our study also has several notable strengths in comparison to previous studies. We utilise the Illumina EPIC array with 850,000 CpG sites, compared to the approximately 450,000 available on the 450 K array used in most previous studies. In our discovery analysis, we require the CpG to associate with the liver variable in at least two independent cohorts and in the meta-analysis involving over 5500 individuals. Furthermore, we are able to investigate the association between the discovered CpGs and liver variables across three different generations and age groups in the multigenerational YFG 2018 follow-up.

## Conclusions

We show that in YFS, methylation at cg06690548 (*SLC7A11*) is lower in individuals with ultrasonically-identified liver steatosis, with this association becoming more pronounced in older individuals. We further identify overlapping groups of CpGs associating with both GGT levels and FLI, many of which also exhibit stronger associations with these liver health indicators in older YFS generations. Although several meQTLs were identified for these CpGs which are linked to liver health, these meQTLs did not affect the associations with liver-related traits, nor were they independently associated with liver health. Interestingly, some of the associations between the identified CpGs and FLI were mediated by whole blood gene expression, suggesting a possible broader systemic connection. In contrast, methylation levels at those CpGs associated with GGT rarely associated with expression of nearby genes, pointing to potentially distinct mechanisms underlying these biomarkers of liver function. Taken together, our findings contribute to a deeper understanding of the pathophysiology of liver diseases and provide insights in the systematic development of steatotic liver and provide information on the crosstalk between the liver and the circulatory immune cells.

## Supplementary Information

Supplementary Material 1.

Supplementary Material 2.

## Data Availability

The datasets generated and/or analyzed during the current study are not publicly available due to restrictions imposed by Finnish and German legislation but are available from the corresponding author/data sharing committees upon a reasonable request.

## Abbreviations

ALD: Alcohol-related liver disease
ALT: Alanine aminotransferase
AST: Aspartate aminotransferase
DMR: Differentially methylated region
eQTM: Expression quantitative trait methylation
FDR: False discovery rate
FLI: Fatty liver index
GGT: Gamma-glutamyltransferase
KORA: Cooperative Health Research in the Region of Augsburg
LURIC: LUdwigshafen RIsk and Cardiovascular Health study
MASLD: Metabolic dysfunction-associated steatotic liver disease
meQTLs: Methylation quantitative trait loci
metALD: Metabolic dysfunction and alcohol-associated liver disease
NAFLD: Non-alcoholic fatty liver disease
PC: Principal component
SLCs: Solute carriers
T2D: Type 2 diabetes
YFS: The Young Finns Study

## References

[1] Rinella ME, Lazarus JV, Ratziu V, Francque SM, Sanyal AJ, Kanwal F, et al. A multisociety Delphi consensus statement on new fatty liver disease nomenclature. Hepatology. 2023;78:1966–86. 37363821 10.1097/HEP.0000000000000520 PMC10653297

[2] Hagström H, Vessby J, Ekstedt M, Shang Y. 99% of patients with NAFLD meet MASLD criteria and natural history is therefore identical. J Hepatol. 2024;80:e76–7. 37678723 10.1016/j.jhep.2023.08.026

[3] Younossi ZM, Golabi P, Paik JM, Henry A, Van Dongen C, Henry L. The global epidemiology of nonalcoholic fatty liver disease (NAFLD) and nonalcoholic steatohepatitis (NASH): a systematic review. Hepatology. 2023;77:1335–47. 36626630 10.1097/HEP.0000000000000004 PMC10026948

[4] Holmer M, Melum E, Isoniemi H, Ericzon B-G, Castedal M, Nordin A, et al. Nonalcoholic fatty liver disease is an increasing indication for liver transplantation in the Nordic countries. Liver Int. 2018;38:2082–90. 29630771 10.1111/liv.13751

[5] Alkhouri N, Hanouneh IA, Zein NN, Lopez R, Kelly D, Eghtesad B, et al. Liver transplantation for nonalcoholic steatohepatitis in young patients. Transplant Int. 2016;29:418–24. 10.1111/tri.12694 26402655

[6] Allen AM, Therneau TM, Larson JJ, Coward A, Somers VK, Kamath PS. Nonalcoholic fatty liver disease incidence and impact on metabolic burden and death: a 20 year-community study. Hepatology. 2018;67:1726. 28941364 10.1002/hep.29546 PMC5866219

[7] Younossi ZM, Koenig AB, Abdelatif D, Fazel Y, Henry L, Wymer M. Global epidemiology of nonalcoholic fatty liver disease—meta-analytic assessment of prevalence, incidence, and outcomes. Hepatology. 2016;64:73. 26707365 10.1002/hep.28431

[8] Li Y, Yang P, Ye J, Xu Q, Wu J, Wang Y. Updated mechanisms of MASLD pathogenesis. Lipids Health Dis. 2024;23:117. 38649999 10.1186/s12944-024-02108-x PMC11034170

[9] Luukkonen PK, Qadri S, Ahlholm N, Porthan K, Männistö V, Sammalkorpi H, et al. Distinct contributions of metabolic dysfunction and genetic risk factors in the pathogenesis of non-alcoholic fatty liver disease. J Hepatol. 2022;76:526–35. 34710482 10.1016/j.jhep.2021.10.013 PMC8852745

[10] Sakurai Y, Kubota N, Yamauchi T, Kadowaki T. Role of insulin resistance in MAFLD. Int J Mol Sci. 2021;22:4156. 33923817 10.3390/ijms22084156 PMC8072900

[11] Ciaula AD, Bonfrate L, Krawczyk M, Frühbeck G, Portincasa P. Synergistic and detrimental effects of alcohol intake on progression of liver steatosis. Int J Mol Sci. 2022;23:2636. 35269779 10.3390/ijms23052636 PMC8910376

[12] Eslam M, Newsome PN, Sarin SK, Anstee QM, Targher G, Romero-Gomez M, et al. A new definition for metabolic dysfunction-associated fatty liver disease: an international expert consensus statement. J Hepatol. 2020;73:202–9. 32278004 10.1016/j.jhep.2020.03.039

[13] Danielsson O, Nano J, Pahkala K, Rospleszcz S, Lehtimäki T, Schlett CL, et al. Validity of fatty liver disease indices in the presence of alcohol consumption. Scand J Gastroenterol. 2022;57:1349–60. 35723012 10.1080/00365521.2022.2085060

[14] Tiffon C. The impact of nutrition and environmental epigenetics on human health and disease. Int J Mol Sci. 2018;19:3425. 30388784 10.3390/ijms19113425 PMC6275017

[15] Lai Z, Chen J, Ding C, Wong K, Chen X, Pu L, et al. Association of hepatic global DNA methylation and serum one-carbon metabolites with histological severity in patients with NAFLD. Obesity (Silver Spring). 2020;28:197–205. 31785086 10.1002/oby.22667

[16] Ahrens M, Ammerpohl O, von Schönfels W, Kolarova J, Bens S, Itzel T, et al. DNA methylation analysis in nonalcoholic fatty liver disease suggests distinct disease-specific and remodeling signatures after bariatric surgery. Cell Metab. 2013;18:296–302. 23931760 10.1016/j.cmet.2013.07.004

[17] Hyun J, Jung Y. DNA methylation in nonalcoholic fatty liver disease. Int J Mol Sci. 2020;21:8138. 33143364 10.3390/ijms21218138 PMC7662478

[18] Nano J, Ghanbari M, Wang W, de Vries PS, Dhana K, Muka T, et al. Epigenome-wide association study identifies methylation sites associated with liver enzymes and hepatic steatosis. Gastroenterology. 2017;153:1096-1106.e2. 28624579 10.1053/j.gastro.2017.06.003

[19] Ma J, Nano J, Ding J, Zheng Y, Hennein R, Liu C, et al. A peripheral blood DNA methylation signature of hepatic fat reveals a potential causal pathway for nonalcoholic fatty liver disease. Diabetes. 2019;68:1073–83. 30936141 10.2337/DB18-1193 PMC6477898

[20] Zeng H, Li W, Xia M, Ge J, Ma H, Chen L, et al. Longitudinal association of peripheral blood DNA methylation with liver fat content: distinguishing between predictors and biomarkers. Lipids Health Dis. 2024;23:309. 39334355 10.1186/s12944-024-02304-9 PMC11429307

[21] Raitakari OT, Juonala M, Ronnemaa T, Keltikangas-Jarvinen L, Rasanen L, Pietikainen M, et al. Cohort profile: the cardiovascular risk in Young Finns Study. Int J Epidemiol. 2008;37:1220–6. 18263651 10.1093/ije/dym225

[22] Pahkala K, Rovio S, Auranen K, Bourgery M, Elovainio M, Fogelholm M, et al. Cohort profile update expanding the Cardiovascular Risk in Young Finns Study into a multigenerational cohort. MedRxiv. 2024. 10.1101/2024.11.24.24317769. 10.1093/ije/dyaf206 41489589

[23] Holle R, Happich M, Lowel H, Wichmann HE, Group MS. KORA–a research platform for population based health research. Gesundheitswesen Bundesverband der Arzte des Offentlichen Gesundheitsdienstes (Germany). 2005; 67 Suppl 1:S19-25. 10.1055/s-2005-858235 16032513

[24] Wichmann HE, Gieger C, Illig T. KORA-gen - resource for population genetics, controls and a broad spectrum of disease phenotypes. Gesundheitswesen. 2005. 10.1055/s-2005-858226. 16032514 10.1055/s-2005-858226

[25] Winkelmann BR, Marz W, Boehm BO, Zotz R, Hager J, Hellstern P, et al. Rationale and design of the LURIC study–a resource for functional genomics, pharmacogenomics and long-term prognosis of cardiovascular disease. Pharmacogenomics. 2001;2:S1-73. 11258203 10.1517/14622416.2.1.S1

[26] Kananen L, Marttila S, Nevalainen T, Kummola L, Junttila I, Mononen N, et al. The trajectory of the blood DNA methylome ageing rate is largely set before adulthood: evidence from two longitudinal studies. Age. 2016;38:65. 27300324 10.1007/s11357-016-9927-9 PMC5005919

[27] Marttila S, Viiri LE, Mishra PP, Kühnel B, Matias-Garcia PR, Lyytikäinen L-P, et al. Methylation status of nc886 epiallele reflects periconceptional conditions and is associated with glucose metabolism through nc886 RNAs. Clin Epigenetics. 2021. 10.1186/s13148-021-01132-3. 34294131 10.1186/s13148-021-01132-3 PMC8296652

[28] Aryee MJ, Jaffe AE, Corrada-Bravo H, Ladd-Acosta C, Feinberg AP, Hansen KD, et al. Minfi: a flexible and comprehensive Bioconductor package for the analysis of Infinium DNA methylation microarrays. Bioinformatics. 2014. 10.1093/bioinformatics/btu049. 24478339 10.1093/bioinformatics/btu049 PMC4016708

[29] McCartney DL, Walker RM, Morris SW, McIntosh AM, Porteous DJ, Evans KL. Identification of polymorphic and off-target probe binding sites on the Illumina Infinium MethylationEPIC BeadChip. Genom Data. 2016;9:22–4. 27330998 10.1016/j.gdata.2016.05.012 PMC4909830

[30] Pidsley R, Zotenko E, Peters TJ, Lawrence MG, Risbridger GP, Molloy P, et al. Critical evaluation of the Illumina MethylationEPIC BeadChip microarray for whole-genome DNA methylation profiling. Genome Biol. 2016;17:208. 27717381 10.1186/s13059-016-1066-1 PMC5055731

[31] Zeilinger S, Kuhnel B, Klopp N, Baurecht H, Kleinschmidt A, Gieger C, et al. Tobacco smoking leads to extensive genome-wide changes in DNA methylation. PLoS ONE. 2013;8:e63812. 23691101 10.1371/journal.pone.0063812 PMC3656907

[32] Laaksonen J, Mishra PP, Seppälä I, Raitoharju E, Marttila S, Mononen N, Lyytikäinen L-P, Kleber ME, Delgado GE, Lepistö M, Almusa H, Ellonen P, Lorkowski S, März W, Hutri-Kähönen N, Raitakari O, Kähönen M, Salonen JT, Lehtimäki T. Mitochondrial genome-wide analysis of nuclear DNA methylation quantitative trait loci. Hum Mol Genet. 2021;ddab339. 10.1093/hmg/ddab339 PMC9122653 35077545

[33] Price ME, Cotton AM, Lam LL, Farré P, Emberly E, Brown CJ, et al. Additional annotation enhances potential for biologically-relevant analysis of the Illumina Infinium HumanMethylation450 BeadChip array. Epigenetics Chromatin. 2013;6:4. 23452981 10.1186/1756-8935-6-4 PMC3740789

[34] Peters TJ, Buckley MJ, Statham AL, Pidsley R, Samaras K, V Lord R, et al. De novo identification of differentially methylated regions in the human genome. Epigenetics Chromatin. 2015;8:6. 25972926 10.1186/1756-8935-8-6 PMC4429355

[35] Du P, Zhang X, Huang C-C, Jafari N, Kibbe WA, Hou L, et al. Comparison of beta-value and M-value methods for quantifying methylation levels by microarray analysis. BMC Bioinformatics. 2010;11:587. 21118553 10.1186/1471-2105-11-587 PMC3012676

[36] Mansell G, Gorrie-Stone TJ, Bao Y, Kumari M, Schalkwyk LS, Mill J, et al. Guidance for DNA methylation studies: statistical insights from the Illumina EPIC array. BMC Genomics. 2019;20:366. 31088362 10.1186/s12864-019-5761-7 PMC6518823

[37] Smith EN, Chen W, Kähönen M, Kettunen J, Lehtimäki T, Peltonen L, et al. Longitudinal genome-wide association of cardiovascular disease risk factors in the Bogalusa heart study. PLoS Genet. 2010;6:e1001094. 20838585 10.1371/journal.pgen.1001094 PMC2936521

[38] Taliun D, Harris DN, Kessler MD, Carlson J, Szpiech ZA, Torres R, Taliun SAG, Corvelo A, Gogarten SM, Kang HM, Pitsillides AN, LeFaive J, Lee S-B, Tian X, Browning BL, Das S, Emde A-K, Clarke WE, Loesch DP, Shetty AC, Blackwell TW, Smith AV, Wong Q, Liu X, Conomos MP, Bobo DM, Aguet F, Albert C, Alonso A, Ardlie KG, Arking DE, Aslibekyan S, Auer PL, Barnard J, Barr RG, Barwick L, Becker LC, Beer RL, Benjamin EJ, Bielak LF, Blangero J, Boehnke M, Bowden DW, Brody JA, Burchard EG, Cade BE, Casella JF, Chalazan B, Chasman DI, Chen Y-DI, Cho MH, Choi SH, Chung MK, Clish CB, Correa A, Curran JE, Custer B, Darbar D, Daya M, de Andrade M, DeMeo DL, Dutcher SK, Ellinor PT, Emery LS, Eng C, Fatkin D, Fingerlin T, Forer L, Fornage M, Franceschini N, Fuchsberger C, Fullerton SM, Germer S, Gladwin MT, Gottlieb DJ, Guo X, Hall ME, He J, Heard-Costa NL, Heckbert SR, Irvin MR, Johnsen JM, Johnson AD, Kaplan R, Kardia SLR, Kelly T, Kelly S, Kenny EE, Kiel DP, Klemmer R, Konkle BA, Kooperberg C, Köttgen A, Lange LA, Lasky-Su J, Levy D, Lin X, Lin K-H, Liu C, Loos RJF, Garman L, Gerszten R, Lubitz SA, Lunetta KL, Mak ACY, Manichaikul A, Manning AK, Mathias RA, McManus DD, McGarvey ST, Meigs JB, Meyers DA, Mikulla JL, Minear MA, Mitchell BD, Mohanty S, Montasser ME, Montgomery C, Morrison AC, Murabito JM, Natale A, Natarajan P, Nelson SC, North KE, O’Connell JR, Palmer ND, Pankratz N, Peloso GM, Peyser PA, Pleiness J, Post WS, Psaty BM, Rao DC, Redline S, Reiner AP, Roden D, Rotter JI, Ruczinski I, Sarnowski C, Schoenherr S, Schwartz DA, Seo J-S, Seshadri S, Sheehan VA, Sheu WH, Shoemaker MB, Smith NL, Smith JA, Sotoodehnia N, Stilp AM, Tang W, Taylor KD, Telen M, Thornton TA, Tracy RP, Van Den Berg DJ, Vasan RS, Viaud-Martinez KA, Vrieze S, Weeks DE, Weir BS, Weiss ST, Weng L-C, Willer CJ, Zhang Y, Zhao X, Arnett DK, Ashley-Koch AE, Barnes KC, Boerwinkle E, Gabriel S, Gibbs R, Rice KM, Rich SS, Silverman EK, Qasba P, Gan W, NHLBI Trans-Omics for Precision Medicine (TOPMed) Consortium, Papanicolaou GJ, Nickerson DA, Browning SR, Zody MC, Zöllner S, Wilson JG, Cupples LA, Laurie CC, Jaquish CE, Hernandez RD, O’Connor TD, Abecasis GR. Sequencing of 53,831 diverse genomes from the NHLBI TOPMed Program. Nature 2021;590:290–299. 10.1038/s41586-021-03205-y PMC7875770 33568819

[39] Elovainio M, Taipale T, Seppala I, Mononen N, Raitoharju E, Jokela M, et al. Activated immune-inflammatory pathways are associated with long-standing depressive symptoms: evidence from gene-set enrichment analyses in the Young Finns Study. J Psychiatr Res. 2015;71:120–5. 26473696 10.1016/j.jpsychires.2015.09.017

[40] Rückert IM, Heier M, Rathmann W, Baumeister SE, Döring A, Meisinger C. Association between markers of fatty liver disease and impaired glucose regulation in men and women from the general population: The KORA-F4-study. PLoS ONE. 2011. 10.1371/journal.pone.0022932. 21850244 10.1371/journal.pone.0022932 PMC3151286

[41] Lerchbaum E, Pilz S, Grammer TB, Boehm BO, Stojakovic T, Obermayer-Pietsch B, et al. The fatty liver index is associated with increased mortality in subjects referred to coronary angiography. Nutr Metab Cardiovasc Dis. 2013;23:1231–8. 23557879 10.1016/j.numecd.2013.02.004

[42] Bedogni G, Kahn HS, Bellentani S, Tiribelli C. A simple index of lipid overaccumulation is a good marker of liver steatosis. BMC Gastroenterol. 2010;10:98. 20738844 10.1186/1471-230X-10-98 PMC2940930

[43] Suomela E, Oikonen M, Virtanen J, Parkkola R, Jokinen E, Laitinen T, Hutri-Kahonen N, Kahonen M, Lehtimaki T, Taittonen L, Tossavainen P, Jula A, Loo BM, Mikkila V, Younossi Z, Viikari JS, Juonala M, Raitakari OT. Prevalence and determinants of fatty liver in normal-weight and overweight young adults. The Cardiovasc Risk Young Finns Stud. Ann Med 2014; 1–7. 10.3109/07853890.2014.966752 25333756

[44] Moissl AP, Delgado GE, Krämer BK, Dawczynski C, Stojakovic T, März W, et al. Alcohol consumption and mortality: the Ludwigshafen Risk and Cardiovascular Health (LURIC) study. Atherosclerosis. 2021;335:119–25. 34454737 10.1016/j.atherosclerosis.2021.08.014

[45] Houseman EA, Accomando WP, Koestler DC, Christensen BC, Marsit CJ, Nelson HH, et al. DNA methylation arrays as surrogate measures of cell mixture distribution. BMC Bioinform. 2012;13:86. 10.1186/1471-2105-13-86 PMC3532182 22568884

[46] van Iterson M, van Zwet EW, Heijmans BT. Controlling bias and inflation in epigenome- and transcriptome-wide association studies using the empirical null distribution. Genome Biol. 2017;18:19. 28129774 10.1186/s13059-016-1131-9 PMC5273857

[47] Willer CJ, Li Y, Abecasis GR. METAL: fast and efficient meta-analysis of genomewide association scans. Bioinformatics. 2010;26:2190–1. 20616382 10.1093/bioinformatics/btq340 PMC2922887

[48] Peters TJ, Buckley MJ, Statham AL, Pidsley R, Samaras K, V Lord R, Clark SJ, Molloy PL. De novo identification of differentially methylated regions in the human genome. Epigenetics & Chromatin. 2015;8:6. 10.1186/1756-8935-8-6 PMC4429355 25972926

[49] Ciantar J, Marttila S, Rajić S, Kostiniuk D, Mishra PP, Lyytikäinen L-P, et al. Identification and functional characterisation of DNA methylation differences between East- and West-originating Finns. Epigenetics. 2024;19:2397297. 39217505 10.1080/15592294.2024.2397297 PMC11382697

[50] Min JL, Hemani G, Hannon E, Dekkers KF, Castillo-Fernandez J, Luijk R, et al. Genomic and phenotypic insights from an atlas of genetic effects on DNA methylation. Nat Genet. 2021;53:1311–21. 34493871 10.1038/s41588-021-00923-x PMC7612069

[51] Chang CC, Chow CC, Tellier LC, Vattikuti S, Purcell SM, Lee JJ. Second-generation PLINK: rising to the challenge of larger and richer datasets. Gigascience. 2015;4:7. 25722852 10.1186/s13742-015-0047-8 PMC4342193

[52] Smedley D, Haider S, Ballester B, Holland R, London D, Thorisson G, et al. BioMart – biological queries made easy. BMC Genomics. 2009;10:22. 19144180 10.1186/1471-2164-10-22 PMC2649164

[53] VanderWeele TJ. Mediation analysis: a practitioner’s guide. Annu Rev Public Health. 2016;37:17–32. 26653405 10.1146/annurev-publhealth-032315-021402

[54] Miles CH. On the causal interpretation of randomised interventional indirect effects. J R Stat Soc Ser B Stat Methodol. 2023;85:1154–72.

[55] Valeri L, Vanderweele TJ. Mediation analysis allowing for exposure-mediator interactions and causal interpretation: theoretical assumptions and implementation with SAS and SPSS macros. Psychol Methods. 2013;18:137–50. 23379553 10.1037/a0031034 PMC3659198

[56] Melton PE, Burton MA, Lillycrop KA, Godfrey KM, Rauschert S, Anderson D, et al. Differential DNA methylation of steatosis and non-alcoholic fatty liver disease in adolescence. Hepatol Int. 2023;17:584–94. 36737504 10.1007/s12072-022-10469-7 PMC9897882

[57] Wahl S, Drong A, Lehne B, Loh M, Scott WR, Kunze S, et al. Epigenome-wide association study of body mass index, and the adverse outcomes of adiposity. Nature. 2017;541:81–6. 28002404 10.1038/nature20784 PMC5570525

[58] Geurts YM, Dugué P-A, Joo JE, Makalic E, Jung C-H, Guan W, et al. Novel associations between blood DNA methylation and body mass index in middle-aged and older adults. Int J Obes (Lond). 2018;42:887–96. 29278407 10.1038/ijo.2017.269

[59] Mendelson MM, Marioni RE, Joehanes R, Liu C, Hedman ÅK, Aslibekyan S, et al. Association of body mass index with DNA methylation and gene expression in blood cells and relations to cardiometabolic disease: a Mendelian randomization approach. PLoS Med. 2017;14:e1002215. 28095459 10.1371/journal.pmed.1002215 PMC5240936

[60] Sayols-Baixeras S, Subirana I, Fernández-Sanlés A, Sentí M, Lluís-Ganella C, Marrugat J, et al. DNA methylation and obesity traits: an epigenome-wide association study. The REGICOR study. Epigenetics. 2017;12:909–16. 29099282 10.1080/15592294.2017.1363951 PMC5788444

[61] Liu J, Carnero-Montoro E, van Dongen J, Lent S, Nedeljkovic I, Ligthart S, et al. An integrative cross-omics analysis of DNA methylation sites of glucose and insulin homeostasis. Nat Commun. 2019;10:2581. 31197173 10.1038/s41467-019-10487-4 PMC6565679

[62] Hillary RF, McCartney DL, Smith HM, Bernabeu E, Gadd DA, Chybowska AD, et al. Blood-based epigenome-wide analyses of 19 common disease states: a longitudinal, population-based linked cohort study of 18,413 Scottish individuals. PLoS Med. 2023;20:e1004247. 37410739 10.1371/journal.pmed.1004247 PMC10325072

[63] Dugué P-A, Wilson R, Lehne B, Jayasekara H, Wang X, Jung C-H, et al. Alcohol consumption is associated with widespread changes in blood DNA methylation: analysis of cross-sectional and longitudinal data. Addict Biol. 2021;26:e12855. 31789449 10.1111/adb.12855

[64] Liu C, Marioni RE, Hedman ÅK, Pfeiffer L, Tsai P-C, Reynolds LM, et al. A DNA methylation biomarker of alcohol consumption. Mol Psychiatry. 2018;23:422–33. 27843151 10.1038/mp.2016.192 PMC5575985

[65] Liang X, Justice AC, So-Armah K, Krystal JH, Sinha R, Xu K. DNA methylation signature on phosphatidylethanol, not on self-reported alcohol consumption, predicts hazardous alcohol consumption in two distinct populations. Mol Psychiatry. 2021;26:2238–53. 32034291 10.1038/s41380-020-0668-x PMC8440221

[66] Xu K, Montalvo-Ortiz JL, Zhang X, Southwick SM, Krystal JH, Pietrzak RH, et al. Epigenome-wide DNA methylation association analysis identified novel loci in peripheral cells for alcohol consumption among European American male veterans. Alcohol Clin Exp Res. 2019;43:2111–21. 31386212 10.1111/acer.14168 PMC9377208

[67] Li T, Yi J, Wu H, Wang K, Zhou B. SLC7A11 in hepatocellular carcinoma: potential mechanisms, regulation, and clinical significance. Am J Cancer Res. 2024;14:2326–42. 38859833 10.62347/KGCL7357 PMC11162675

[68] Liu Y, Wu K, Fu Y, Li W, Zhao X-Y. Slc7a11 stimulates glutathione synthesis to preserve fatty acid metabolism in primary hepatocytes. Redox Rep. 2023;28:2260646. 37750478 10.1080/13510002.2023.2260646 PMC10540662

[69] Vallerga CL, Zhang F, Fowdar J, McRae AF, Qi T, Nabais MF, et al. Analysis of DNA methylation associates the cystine–glutamate antiporter SLC7A11 with risk of Parkinson’s disease. Nat Commun. 2020;11:1238. 32144264 10.1038/s41467-020-15065-7 PMC7060318

[70] Zhu Z, Zhang F, Hu H, Bakshi A, Robinson MR, Powell JE, et al. Integration of summary data from GWAS and eQTL studies predicts complex trait gene targets. Nat Genet. 2016;48:481–7. 27019110 10.1038/ng.3538

[71] Mosquera OA. Hidden among the crowd: differential DNA methylation-expression correlations in cancer occur at important oncogenic pathways. Front Genet. 2015;6:163. 10.3389/fgene.2015.00163. 26029238 10.3389/fgene.2015.00163 PMC4429616

[72] Simner C, Novakovic B, Lillycrop KA, Bell CG, Harvey NC, Cooper C, et al. DNA methylation of amino acid transporter genes in the human placenta. Placenta. 2017;60:64–73. 29208242 10.1016/j.placenta.2017.10.010 PMC6049612

[73] Neve B, Fernandez-Zapico ME, Ashkenazi-Katalan V, Dina C, Hamid YH, Joly E, et al. Role of transcription factor KLF11 and its diabetes-associated gene variants in pancreatic beta cell function. Proc Natl Acad Sci USA. 2005;102:4807–12. 15774581 10.1073/pnas.0409177102 PMC554843

[74] Arpón A, Milagro FI, Ramos-Lopez O, Mansego ML, Santos JL, Riezu-Boj J-I, et al. Epigenome-wide association study in peripheral white blood cells involving insulin resistance. Sci Rep. 2019;9:2445. 30792424 10.1038/s41598-019-38980-2 PMC6385280

[75] Hillary RF, Ng HK, McCartney DL, Elliott HR, Walker RM, Campbell A, et al. Blood-based epigenome-wide analyses of chronic low-grade inflammation across diverse population cohorts. Cell Genom. 2024;4:100544. 38692281 10.1016/j.xgen.2024.100544 PMC11099341

[76] Karabegović I, Portilla-Fernandez E, Li Y, Ma J, Maas SCE, Sun D, et al. Epigenome-wide association meta-analysis of DNA methylation with coffee and tea consumption. Nat Commun. 2021;12:2830. 33990564 10.1038/s41467-021-22752-6 PMC8121846

[77] Hedman ÅK, Mendelson MM, Marioni RE, Gustafsson S, Joehanes R, Irvin MR, et al. Epigenetic patterns in blood associated with lipid traits predict incident coronary heart disease events and are enriched for results from genome-wide association studies. Circ Cardiovasc Genet. 2017;10:e001487. 28213390 10.1161/CIRCGENETICS.116.001487 PMC5331877

