# Supplementary material for: Genome-wide DNA methylation patterns for indicators of liver steatosis: a longitudinal multiomic study
Source: Clin Epigenetics. 2026 Jan 3;18:22. doi: 10.1186/s13148-025-02037-1 (PMC12866020; doi:10.1186/s13148-025-02037-1)
Supplement: Supplementary file 2 — Supplementary Material 2. [file 13148_2025_2037_MOESM2_ESM.pdf]

Supplementary Figures

Title: Genome-wide DNA methylation patterns for indicators of liver steatosis – A longitudinal multiomic study

Jo Ciantar[#], Sonja Rajić[#], Daria Kostiniuk, Ella Raulamo, Noora Kartiosuo, Liye Lai, Pashupati P Mishra, Leo-Pekka Lyytikäinen, Marcus E Kleber, Suvi Rovio, Juha Mykkänen, Katja Pahkala, Annette Peters, Juliane Winkelmann, Winfried März, Mika Kähönen, Olli Raitakari, Terho Lehtimäki, Melanie Waldenberger, Saara Marttila, Emma Raitoharju*

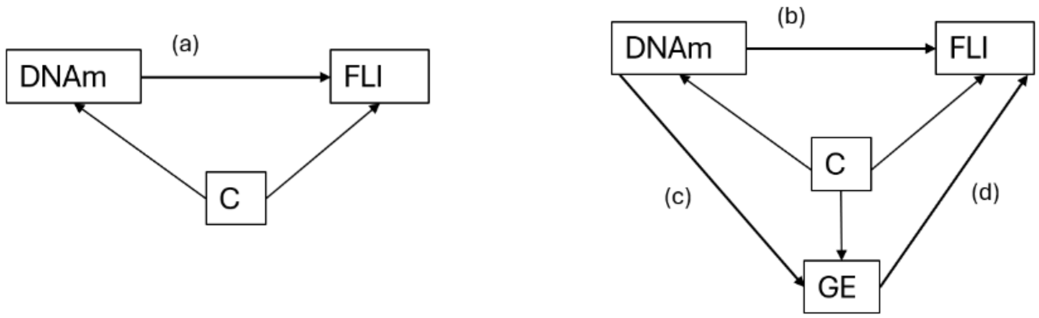

Supplementary Figure 1. The assumed causal associations of the mediation question presented as two directed acyclic graphs. A) describes the total effect, i.e., the effect of DNA methylation on FLI, adjusted for confounders denoted by C, while B) includes also the mediator (gene expression), assumed to be affected by DNA methylation and subsequently to affect FLI.

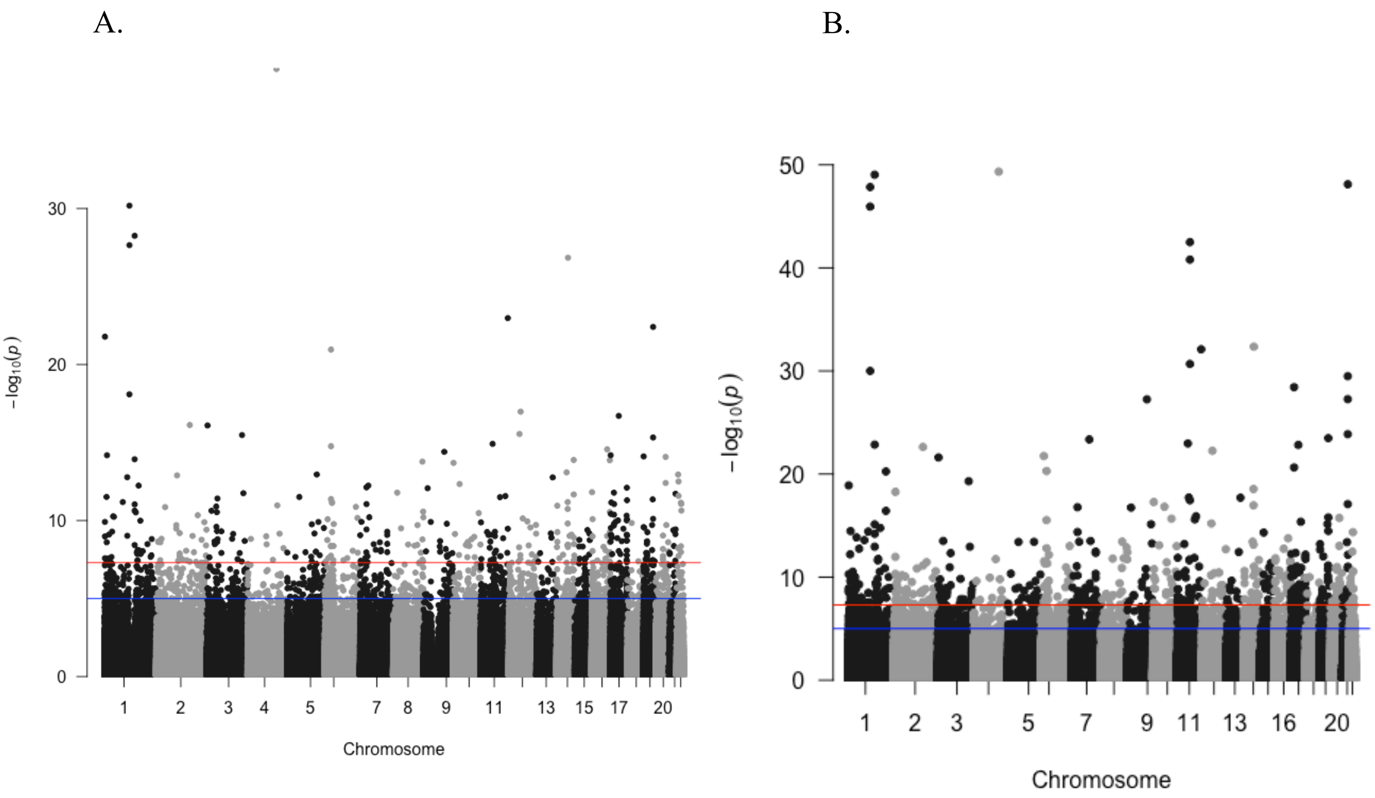

Supplementary Figure 2. Manhattan plots created from the META analysis results of DNA whole blood DNA methylation levels and A) GGT levels and B) FLI levels. Similar to LURIC results, META analysis results are inflated (A)λ for GGT =1.47 and B) λ for FLI is 1.48) and thus these meta-analysis results were not used to select sites for further investigation.
